# Effect and cost-effectiveness of educating mothers about childhood DPT vaccination on immunisation uptake, knowledge, and perceptions in Uttar Pradesh, India: A randomised controlled trial

**DOI:** 10.1371/journal.pmed.1002519

**Published:** 2018-03-06

**Authors:** Timothy Powell-Jackson, Camilla Fabbri, Varun Dutt, Sarah Tougher, Kultar Singh

**Affiliations:** 1 Department of Global Health and Development, London School of Hygiene & Tropical Medicine, London, United Kingdom; 2 Sambodhi Research and Communications, Noida, Uttar Pradesh, India; Makerere University Medical School, UGANDA

## Abstract

**Background:**

To assess the effect of health information on immunisation uptake in rural India, we conducted an individually randomised controlled trial of health information messages targeting the mothers of unvaccinated or incompletely vaccinated children through home visits in rural Uttar Pradesh, India.

**Methods and findings:**

The study tested a brief intervention that provided mothers face-to-face with information on the benefits of the tetanus vaccine. Participants were 722 mothers of children aged 0–36 months who had not received 3 doses of diphtheria–pertussis–tetanus (DPT) vaccine (DPT3). Mothers were randomly assigned in a ratio of 1:1:1 to 1 of 3 study arms: mothers in the first treatment group received information framed as a gain (e.g., the child is less likely to get tetanus and more likely to be healthy if vaccinated), mothers in the second treatment group received information framed in terms of a loss (e.g., the child is more likely to get tetanus and suffer ill health if not vaccinated), and the third arm acted as a control group, with no information given to the mother. Surveys were conducted at baseline (September 2015) and after the intervention (April 2016). The primary outcome was the proportion of children who had received DPT3 measured after 7 months of follow-up. The analysis was by intention to treat. A total of 16 (2.2%) participants were lost to follow-up. The coverage of DPT3 was 28% in the control group and 43% in the pooled information groups, giving a risk difference of 15 percentage points (95% CI: 7% to 22%, *p <* 0.001) and a relative risk of 1.52 (95% CI: 1.2 to 1.9, *p <* 0.001). The information intervention increased the rate of measles vaccination by 22 percentage points (risk difference: 22%, 95% CI: 14% to 30%, *p <* 0.001; relative risk: 1.53, 95% CI: 1.29 to 1.80) and the rate of full immunisation by 14 percentage points (risk difference: 14%, 95% CI: 8% to 21%, *p <* 0.001; relative risk: 1.72, 95% CI: 1.29 to 2.29). It had a large positive effect on knowledge of the causes, symptoms, and prevention of tetanus but no effect on perceptions of vaccine efficacy. There was no difference in the proportion of children with DPT3 between the group that received information framed as a loss and the group that received information framed as a gain (risk difference: 4%, 95% CI: −5% to 13%; *p* = 0.352; relative risk: 1.11, 95% CI: 0.90 to 1.36). The cost per disability-adjusted life year averted of providing information was US$186, making the intervention highly cost-effective with respect to the WHO-recommended threshold of once the gross domestic product per capita (US$793 in the case of Uttar Pradesh). Key study limitations include the modest sample size for this trial, limiting power to detect small differences in the framing of information, and the potential for contamination among households.

**Conclusions:**

Providing mothers of unvaccinated/incompletely vaccinated children with information on tetanus and the benefits of DPT vaccination substantially increased immunisation coverage and was highly cost-effective. The framing of the health information message did not appear to matter.

**Trial registration:**

The trial is registered with ISRCTN, number ISRCTN84560580.

## Abstract 2

In an individually randomised trial, Timothy Powell-Jackson and colleagues assess the effectiveness of providing health information through home visits to mothers of unvaccinated children in rural Uttar Pradesh, India.

## Author summary

**Why was this study done?:**

- Only 51% of children aged 12–23 months are fully immunised in Uttar Pradesh, an Indian state of over 200 million people.
- Few studies have evaluated the effect and cost-effectiveness of health information messages to improve the uptake of childhood vaccinations, and whether the framing of the information has implications for outcomes.

**What did the researchers do and find?:**

- We conducted an individually randomised controlled trial to test a brief intervention that provided mothers through home visits with health information messages on tetanus and the benefits of the diphtheria–pertussis–tetanus (DPT) vaccine, involving 722 mothers of children aged 0 to 36 months in Uttar Pradesh.
- We framed the health information messages in 2 alternative ways: as a gain—e.g., the child is more likely to be healthy if vaccinated—and as a loss—e.g., the child is more likely suffer ill health if not vaccinated.
- Seven months later, children whose mothers received the information were 52% more likely to have received 3 doses of DPT vaccine than children in the control group, but there was no effect of framing.
- The cost per disability-adjusted life year averted was US$186, making the intervention highly cost-effective with respect to the WHO-recommended threshold of once the gross domestic product per capita (US$793 in the case of Uttar Pradesh).

**What do these findings mean?:**

- The study suggests that providing mothers of unvaccinated/incompletely vaccinated children with health information messages on the benefits of vaccination can be a highly cost-effective means of improving child health through increases in immunisation coverage.

## Introduction

An estimated 5.9 million children die each year globally, of which 1.2 million are in India [1]. The majority of these deaths are preventable with existing low-cost health technologies, such as improved water and sanitation, zinc supplementation, oral rehydration solutions, and vaccines [2]. Indeed, such interventions have contributed to remarkable improvements in child mortality in many developing countries [1]. Despite well-documented evidence on the health and developmental benefits of immunisation [3], a huge number of children fail to get vaccinated. In Uttar Pradesh, a state of more than 200 million people and the setting for this study, only 51% of children aged 12 to 23 months are fully vaccinated [4].

There are many potential reasons for inadequate levels of immunisation coverage. They include problems in the supply of vaccines as well as demand-side factors such as time costs, high discount rates, distrust, fear, and limited knowledge [5,6]. If parents underestimate the true efficacy of vaccines or are simply unaware of their existence, it is plausible that providing information could increase uptake of vaccinations. There is reason to believe that parents may be poorly informed as to the benefits of immunisation. Female literacy is far from universal in many in low- and middle-income countries (LMICs), and information problems are likely to be pervasive. Accurately inferring the risk of disease and protective effect of vaccines through observations in daily life is unrealistic. In fact, provision of health information and education to parents and community members is commonly used to try to stimulate uptake for health interventions in LMICs. Information interventions have the benefit of being low cost to implement, and their effects are thought to be sustainable if they succeed in changing behaviours. For health interventions, such as vaccinations, even short-lived changes in behaviour could lead to large health impacts because once vaccinated, the child is immunised for years to come.

A range of interventions to increase uptake of childhood immunisation in LMICs have been studied, including monetary incentives, health information and education, training of providers, outreach sessions, and home visits [7–9]. However, the handful of studies that have tested the effect of information shed little light on how information increases coverage of immunisation, its cost-effectiveness, and whether the framing of information has implications for outcomes. With the introduction of relatively new vaccines, to prevent pneumococcal pneumonia and rotavirus diarrhoea, and lagging coverage in routine vaccinations, the need for rigorous research on cost-effective ways to increase uptake of childhood vaccinations is urgently needed.

This study reports findings from a randomised trial that examined the extent to which health information messages designed to educate mothers on the benefits of the combined diphtheria–pertussis–tetanus (DPT) vaccine increased immunisation coverage.

## Methods

### Ethical approval

The study received ethical approval from the Indian Council of Medical Research (HMSC/2014/10/HSR), the Public Healthcare Society in India (10/Nov/2013), and the London School of Hygiene & Tropical Medicine in the UK (8610). Mothers gave written informed consent to participate in the study. The trial is registered with ISRCTN (ISRCTN84560580).

### Study design and participants

We undertook a 3-arm randomised controlled trial to evaluate the effect of a brief information intervention on the uptake of vaccination services in 180 villages (clusters) across 6 districts of Uttar Pradesh, India, between 12 September 2015 and 29 April 2016 (S1 Text). The districts and villages were selected as part of a broader research project in which this study was embedded; these sampling procedures are described elsewhere (S2 Text) [10]. Data from the Indian Census 2011 suggest that the study clusters were demographically similar to the study districts and the state (S1 Fig).

Mothers were the intended recipient of the information intervention. They were eligible for inclusion in the study if their child was alive, was aged 0–36 months, and had not received 3 doses of DPT vaccine (DPT3) and if the mother intended to remain in the study area for at least 6 months. Eligible mothers were randomly assigned in a ratio of 1:1:1 to 1 of 3 study arms: mothers in the first treatment group received information framed as a gain, mothers in the second treatment group received information framed in terms of a loss, and the third arm acted as a control group, with no information given to the mother. A computer random number generator in CSPro (version 6.1) assigned participants to 1 of the 3 study arms during the baseline visit. The sequencing of events during the household visit was as follows: mothers were invited to give consent to participate in the study, they were interviewed for the baseline survey, they were assigned to treatment or control, and, if assigned to the treatment group, they received the information intervention.

Potentially eligible participants were identified from lists of mothers generated using 2 sources of information: (i) a representative household maternal and child health survey of 3,600 mothers conducted by the research team in the same villages 9 months prior to the start of this study and (ii) a list of mothers who had given birth in the past year provided by the community health worker (accredited social health activist [ASHA]) in each village. The ASHA in each village was identified by field staff through a home visit during the baseline survey. Field staff visited the household of each potentially eligible mother and assessed eligibility based on either vaccination cards or self-reports. A total of 722 eligible mothers were identified—459 through the previous household survey and 263 using the lists provided by ASHAs.

### Intervention

The study tested an information intervention that provided mothers with information on the benefits of the tetanus vaccine. The intervention was implemented by Sambodhi Research and Communications, a research organisation in Uttar Pradesh. Field staff were mostly male, had completed secondary school, and were from the same state but were not known to the communities. The information was delivered to mothers face-to-face in the privacy of their home, and field staff followed a script that they were trained to deliver in a standardised manner. Specifically, it described the causes and symptoms of tetanus, possible health consequences, the individual benefit of the combination DPT vaccine in terms of mortality and morbidity gains, and the wider community benefits associated with herd immunity.

There were 2 versions of the script that differed in the way the information was framed. The first framed the information on tetanus vaccination as gains—e.g., the child is less likely to get tetanus and more likely to be healthy if vaccinated. The second framed information on tetanus vaccination as a loss—e.g., the child is more likely to get tetanus and suffer ill health if not vaccinated. Visual aids were used to help convey the information in an accessible manner to illiterate women, and a Hindi leaflet containing the information was left with the mother (S2 and S3 Figs). A short question and answer session followed the provision of the information to ensure comprehension of the information. The intervention took about 10 minutes to deliver. The intervention design was informed by the theory behind framing [11–14], previous research on framing in health [15], and extensive piloting.

Both variants of the intervention informed mothers where in the public sector they could get their child vaccinated. The Indian Academy of Pediatrics recommends that 3 doses of DPT should be given, at 6 weeks, 10 weeks, and 14 weeks. The minimum age for this vaccine is 6 weeks. If any of these doses are missed, then the recommended age for catch-up is any time up to 7 years [16]. All study participants were asked questions during the baseline survey on the vaccination status of their child.

### Data collection

Data were collected at baseline in September 2015 and 7 months later at endline in April 2016. At baseline, 722 eligible mothers were interviewed. The survey tools were designed to capture the immunisation status of the child and the mother’s knowledge of the causes of, symptoms of, and prevention methods against tetanus. Immunisation status was assessed in the standard way using the vaccination card and, if not available, self-reports from the mother. The interview also included ‘games’ with chickpeas designed to elicit women’s perceptions of the efficacy of tetanus and measles vaccination, as well as several verification questions to test comprehension of these games. Data were collected on tablets using computer-assisted personal interviewing. Field staff were blinded to group assignment in the endline survey.

### Outcomes

The pre-specified primary outcome was the proportion of children who had received DPT3 measured after 7 months of follow-up. Pre-specified secondary outcomes were the proportion of children fully vaccinated against tuberculosis, diphtheria, pertussis, tetanus, and measles; the mother’s knowledge of any symptom of tetanus; and the mother’s perception of the efficacy of tetanus vaccination. To understand better the effect of the intervention and how it worked, the secondary outcomes also included measures that were not pre-specified in the study protocol but were planned for prior to data analysis: the proportion of children with measles vaccination, the proportion of children with Bacillus Calmette–Guérin (BCG) vaccination, the mother’s knowledge of any cause of tetanus, and her knowledge of any tetanus prevention method.

Immunisation status was measured using vaccination cards, where available, or self-reports by mothers. Perceptions of the efficacy of the tetanus vaccine were obtained using interactive games, in which women were asked hypothetical questions on the chances of children in 2 villages being infected under different immunisation coverage scenarios. Chickpeas were used to elicit responses between 0 and 10, with 0 corresponding to a perception of 0% efficacy of the vaccine and 10 corresponding to 100% efficacy (S3 Text). Knowledge outcomes on causes, symptoms, and prevention methods were defined as binary variables where success corresponded to knowledge of at least 1 of the possible causes, symptoms, and prevention methods, respectively. Given the nature of the intervention, we did not anticipate, nor did we measure, any adverse events.

### Statistical analysis

To assess balance at baseline, we compared the characteristics of participants across the 3 study groups. We then analysed the effect of the interventions by intention to treat using 2 pre-specified approaches. The first was a pooled analysis in which participants in the 2 intervention arms were grouped together and compared with those in the control group. The second was a treatment group analysis in which each intervention group was compared with the control group and with each other. For binary outcomes, we report the proportion in each group, the difference in proportions between groups, and the risk ratio. For continuous outcomes, we report the mean in each group and the difference in means between groups. Adjusted absolute differences were estimated using ordinary least squares linear regression of the outcome on treatment group assignment indicator(s), controlling for age of the child in months and the outcome at baseline.

We conducted several further analyses. With the exception of the first, these analyses were unplanned and were conducted after the analysis of the pre-specified outcomes. First, we studied the presence of information spillovers by exploiting random variation in the geographical density of households assigned to the treatment groups [17]. Information on the geographical coordinates of households collected during baseline was used to generate our variable of interest, measuring for each household the share (proportion) of other study households within a given radius (250 m, 500 m, and 1 km) that received the information intervention.

Second, we hypothesised that information would increase perceptions of efficacy most for those with inaccurate perceptions at baseline. We compared the mean in our measure of perception of efficacy between treatment and control for 2 subgroups of women, with baseline perception of efficacy above and below the 50% threshold. This allowed us to assess the effect of information on mothers with an inaccurate perception of the efficacy of the tetanus vaccine at baseline versus the effect on mothers with a more accurate perception of efficacy.

Third, we carried out a cost-effectiveness analysis based on effect estimates from the pooled analysis. We considered costs from a provider perspective only [18]. Costs were collected through project accounting systems and categorised as start-up or implementation costs. Costs related to research activities were not included. Calculations were based on 2 scenarios: (i) actual costs within the study and (ii) the cost of scale-up to reach the entire target population in the 6 study districts. The scale-up scenario made no changes to average costs, except that we assumed almost double the number of households could be reached each day if the intervention were delivered outside the confines of a research study. To calculate child deaths averted, we used estimates of the effectiveness of DPT3 and measles vaccines on disease-specific mortality [19,20]. Various sources provided estimates of the proportion of under-5 mortality caused by tetanus, pertussis, and measles [21,22]. We did not consider morbidity benefits, positive externalities (herd immunity), or the fact that more than 1 child in a household may have benefited. We calculated disability-adjusted life years (DALYs) by using a period life expectancy of 67.8 years at 2 years of age from the Indian Census 2011 [23] and applying a discount rate of 3% to future years of life. More details on the assumptions and underlying data are provided in S4 Text. All costs are reported in US dollars.

All analyses were conducted using Stata version 14.2 and ArcGIS 10.3. The primary analysis was done blinded to treatment group assignment.

We conducted sample size calculations recognising that the achievable sample size would be constrained by the prior decision to work in the 180 study villages. In the pooled analysis, we estimated that a sample of 465 participants (155 control, 310 pooled treatment) would provide 80% power to detect an absolute difference of 10 percentage points in the proportion of children with DPT3, assuming a 5% level of significance and a DPT3 vaccination rate of 10% in the control group. In the treatment group analysis, we estimated that a sample of 882 participants (294 in each group) would provide 80% power to detect an absolute difference of 10 percentage points in the rate of DPT3 vaccination between any 2 of the treatment groups, assuming a 5% level of significance and a DPT3 vaccination rate of 20% in the comparison group.

## Results

**Fig 1:**
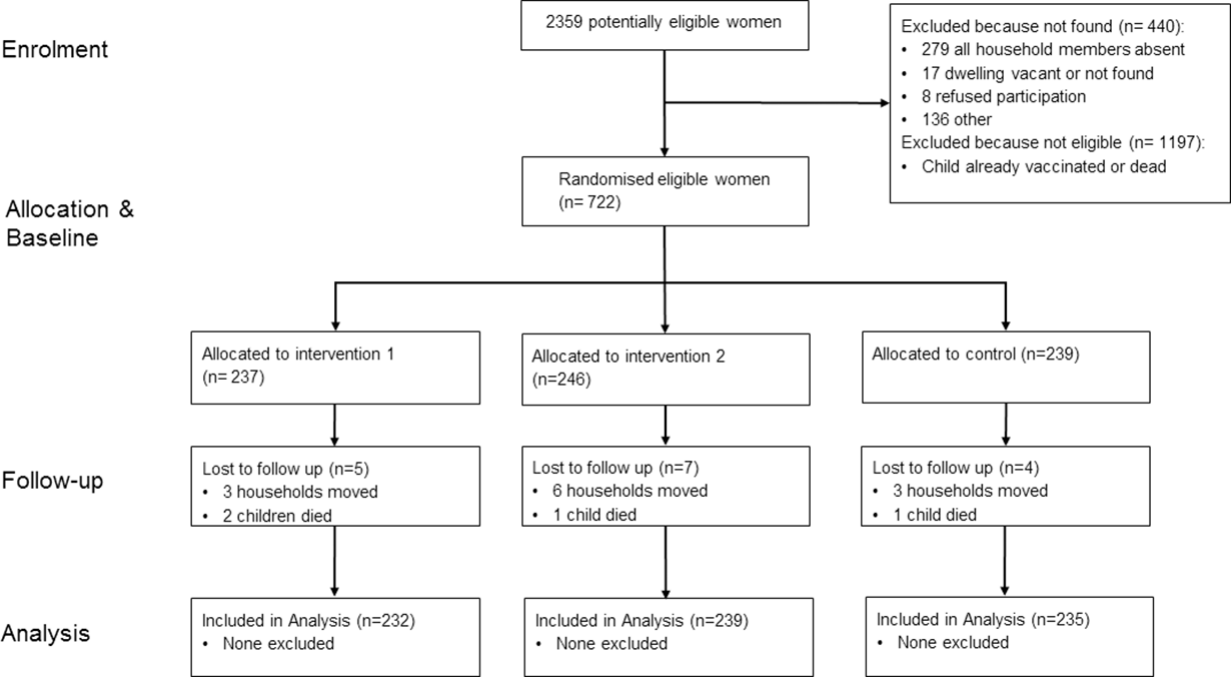
Trial flow of study participants.

Between 12 and 30 July 2015, 2,359 mothers were assessed for eligibility (Fig 1). Of these, 1,637 (69%) mothers were excluded, most commonly because the child had already received DPT3. Overall, 722 participants were enrolled and randomly assigned to 1 of the 3 treatment groups: information positively framed (*n =* 237), information negatively framed (*n =* 246), or no information (*n =* 239). A total of 16 (2.2%) participants were lost to follow-up, resulting in a final analytical sample of 706. There were no further missing data. Attrition was similar across treatment groups.

Table 1 presents baseline characteristics of the study sample by treatment group. The mean age of children was 10 months. Two-thirds of children had had their first dose of DPT, and measles vaccine coverage was 16%. Two out of 5 mothers could identify at least 1 cause of tetanus and 1 method of prevention; however, less than 1 in 10 mothers were able to identify any of the symptoms. Mothers underestimated the efficacy of the tetanus vaccine—they believed the tetanus vaccine to have an average efficacy of 70% while actual efficacy is around 95%. Participants in the 3 groups were similar in terms of child age, child vaccination coverage, knowledge, perceptions of efficacy, and access to health facilities. For a subset of the sample (*n =* 459), we have a richer set of socioeconomic characteristics from the household survey conducted 9 months prior to the start of the trial. These data also suggest the treatment arms were well balanced (S1 Table).

**Table 1:** Baseline characteristics by treatment group.

Variable	Treatment group		
	Control	Positive framing	Negative framing
Age of child (months)	10.20 (0.48)	10.56 (0.50)	10.06 (0.51)
Child vaccines			
DPT1	165/239 (69%)	152/237 (64%)	153/246 (62%)
DPT2	111/239 (46%)	91/237 (38%)	97/246 (39%)
DPT3	0/239 (0%)	0/237 (0%)	0/246 (0%)
BCG vaccine	210/239 (88%)	195/237 (82%)	204/246 (83%)
Measles vaccine	40/239 (17%)	39/237 (16%)	37/246 (15%)
Mother knows a cause of tetanus	111/239 (46%)	95/237 (40%)	105/246 (43%)
Mother knows a symptom of tetanus	20/239 (8%)	17/237 (7%)	24/246 (10%)
Mother knows a prevention method of tetanus	100/239 (42%)	95/237 (40%)	99/246 (40%)
Perception of tetanus vaccination efficacy (index)	7.38 (0.12)	7.15 (0.13)	7.23 (0.13)
Closest health facility to household			
Government anganwadi centre	137/239 (57%)	153/237 (65%)	156/246 (63%)
Government subcentre	24/239 (10%)	20/237 (8%)	33/246 (13%)
Government clinic	24/239 (10%)	18/237 (8%)	20/246 (8%)
Government other	20/239 (8%)	12/237 (5%)	8/246 (3%)
Private clinic	12/239 (5%)	10/237 (4%)	7/246 (3%)

Values are *n/N* (%) for binary outcomes and mean (standard deviation) for continuous outcomes.

BCG, Bacillus Calmette–Guérin; DPT1, 1 dose of diphtheria–pertussis–tetanus vaccine; DPT2, 2 doses of diphtheria–pertussis–tetanus vaccine; DPT3, 3 doses of diphtheria–pertussis–tetanus vaccine.

The results of the pooled analysis are shown in Table 2. The proportion of children with DPT3 was 28% in the control group and 43% in the 2 groups receiving information, giving a difference of 14.6 percentage points (95% CI: 7.3 to 21.9, *p <* 0.001) and a relative risk of 1.5 (95% CI: 1.2 to 1.9, *p <* 0.001). In other words, children whose mothers received the information were 52% more likely to receive DPT3 than children in the control group. There was a positive effect on DPT3 vaccination regardless of the source of data, suggesting that self-reports were not driven by social desirability bias.

**Table 2:** Effect of information on vaccination uptake and other outcomes (pooled analysis).

Outcome	Control	Treatment	Treatment versus control	
			Difference (95% CI)	Relative risk (95% CI)
**Primary outcome**				
DPT3 vaccination	66/235 (28%)	201/471 (43%)	0.146 (0.07 to 0.22)	1.52 (1.21 to 1.91)
From vaccination card	49/102 (48%)	131/201 (65%)	0.171 (0.05 to 0.29)	1.36 (1.08 to 1.70)
Self-reported	17/133 (13%)	70/270 (26%)	0.131 (0.05 to 0.21)	2.03 (1.25 to 3.30)
**Secondary outcomes**				
Full vaccination	47/235 (20%)	162/471 (34%)	0.144 (0.08 to 0.21)	1.72 (1.29 to 2.29)
BCG vaccination	221/235 (94%)	435/471 (92%)	−0.017 (−0.06 to 0.02)	0.98 (0.94 to 1.02)
Measles vaccination	98/235 (42%)	300/471 (64%)	0.220 (0.14 to 0.27)	1.53 (1.29 to 1.80)
Knowledge of tetanus causes	116/235 (49%)	403/471 (86%)	0.362 (0.29 to 0.43)	1.73 (1.52 to 1.98)
Knowledge of tetanus symptoms	37/235 (16%)	256/471 (54%)	0.386 (0.32 to 0.45)	3.45 (2.54 to 4.69)
Knowledge of tetanus prevention	124/235 (53%)	367/471 (78%)	0.252 (0.18 to 0.33)	1.48 (1.30 to 1.68)
Perception of tetanus vaccination efficacy	7.75 (0.14)	7.92 (0.10)	0.170 (−0.17 to 0.51)	—

Values for the descriptive statistics are *n/N* (%) for binary outcomes and mean (standard deviation) for continuous outcomes.

BCG, Bacillus Calmette–Guérin; DPT3, 3 doses of diphtheria–pertussis–tetanus vaccine.

Results for the secondary outcomes follow a similar pattern (Table 2). The effect of the information intervention on the proportion of children fully immunised was an increase of 14 percentage points (95% CI: 7.7 to 21.1, *p <* 0.001), equivalent to a relative risk of 1.7 (95% CI: 1.3 to 2.3, *p <* 0.001). Despite the information never mentioning measles, the effect on measles coverage was 22 percentage points (95% CI: 14.3 to 29.6, *p <* 0.001). There was no measurable effect on BCG vaccination coverage. In terms of possible pathways, the information intervention had a large positive effect on knowledge of causes, symptoms, and prevention of tetanus. We did not find an effect on perceptions of efficacy (difference: 0.17, 95% CI: −0.17 to 0.51, *p* = 0.318).

Table 3 reports results from the treatment group analysis to determine whether the framing of information mattered. The effect of positive framing on the proportion of children with DPT3 was an increase of 12.4 percentage points compared to control (95% CI: 3.9 to 21.0, *p =* 0.005), compared with an increase of 16.7 percentage points (95% CI: 8.2 to 25.2, *p <* 0.001) when the information was negatively framed. However, there was no difference when the 2 information groups were compared with each other (95% CI: −5 to 13, *p =* 0.352). Looking across all the secondary outcomes, the framing of information had no effect, with the exception of knowledge of prevention. Results remained qualitatively the same when we adjusted estimates with the inclusion of covariates (S2 Table) and when we used a Bonferroni correction to deal with the problem of multiple hypothesis testing (S3 Table).

**Table 3:** Effect of the framing of information (treatment group analysis).

Outcome	Control	Positive	Negative	Difference (95% CI)			Relative risk (95% CI)		
				Pos versus C	Neg versus C	Neg versus Pos	Pos versus C	Neg versus C	Neg versus Pos
**Primary outcome**									
DPT3 vaccination	66/235 (28%)	94/232 (41%)	107/239 (45%)	0.124 (0.04 to 0.21)	0.167 (0.08 to 0.25)	0.043 (−0.05 to 0.13)	1.44 (1.12 to 1.87)	1.59 (1.24 to 2.04)	1.11 (0.90 to 1.36)
From vaccination card	49/102 (48%)	61/97 (63%)	70/104 (67%)	0.148 (0.01 to 0.29)	0.193 (0.06 to 0.33)	0.044 (−0.09 to 0.18)	1.31 (1.02 to 1.69)	1.40 (1.10 to 1.79)	1.07 (0.87 to 1.31)
Self-reported	17/133 (13%)	33/135 (24%)	37/135 (27%)	0.117 (0.03 to 0.21)	0.146 (0.05 to 0.24)	0.030 (−0.08 to 0.13)	1.91 (1.12 to 3.26)	2.14 (1.27 to 3.61)	1.12 (0.75 to 1.68)
**Secondary outcomes**									
Full vaccination	47/235 (20%)	75/232 (32%)	87/239 (36%)	0.123 (0.04 to 0.20)	0.164 (0.08 to 0.24)	0.041 (−0.05 to 0.13)	1.62 (1.18 to 2.22)	1.82 (1.34 to 2.47)	1.13 (0.88 to 1.45)
BCG vaccination	221/235 (94%)	213/232 (92%)	222/239 (93%)	−0.022 (−0.07 to 0.02)	−0.012 (−0.06 to 0.03)	0.011 (−0.04 to 0.06)	0.98 (0.93 to 1.03)	0.99 (0.94 to 1.04)	1.01 (0.96 to 1.07)
Measles vaccination	98/235 (42%)	147/232 (63%)	153/239 (64%)	0.217 (0.13 to 0.31)	0.223 (0.14 to 0.31)	0.007 (−0.08 to 0.09)	1.52 (1.27 to 1.82)	1.54 (1.28 to 1.84)	1.01 (0.88 to 1.16)
Knowledge of tetanus causes	116/235 (49%)	194/232 (84%)	209/239 (87%)	0.343 (0.26 to 0.42)	0.381 (0.30 to 0.46)	0.038 (−0.03 to 0.10)	1.69 (1.47 to 1.95)	1.77 (1.54 to 2.03)	1.05 (0.97 to 1.13)
Knowledge of tetanus symptoms	37/235 (16%)	124/232 (53%)	132/239 (55%)	0.377 (0.30 to 0.46)	0.395 (0.32 to 0.47)	0.018 (−0.07 to 0.11)	3.40 (2.47 to 4.67)	3.51 (2.56 to 4.82)	1.03 (0.88 to 1.22)
Knowledge of tetanus prevention	124/235 (53%)	169/232 (73%)	198/239 (83%)	0.201 (0.12 to 0.29)	0.301 (0.22 to 0.38)	0.100 (0.03 to 0.18)	1.38 (1.20 to 1.60)	1.57 (1.37 to 1.80)	1.14 (1.03 to 1.25)
Perception of tetanus vaccination efficacy	7.75 (0.14)	7.78 (0.14)	8.05 (0.14)	0.031 (−0.36 to 0.42)	0.305 (−0.08 to 0.69)	0.274 (−0.12 to 0.66)	—	—	—

Values for the descriptive statistics are *n/N* (%) for binary outcomes and mean (standard deviation) for continuous outcomes.

BCG, Bacillus Calmette–Guérin; C, control; DPT3, 3 doses of diphtheria–pertussis–tetanus vaccine; Neg, negative framing; Pos, positive framing.

**Fig 2:**
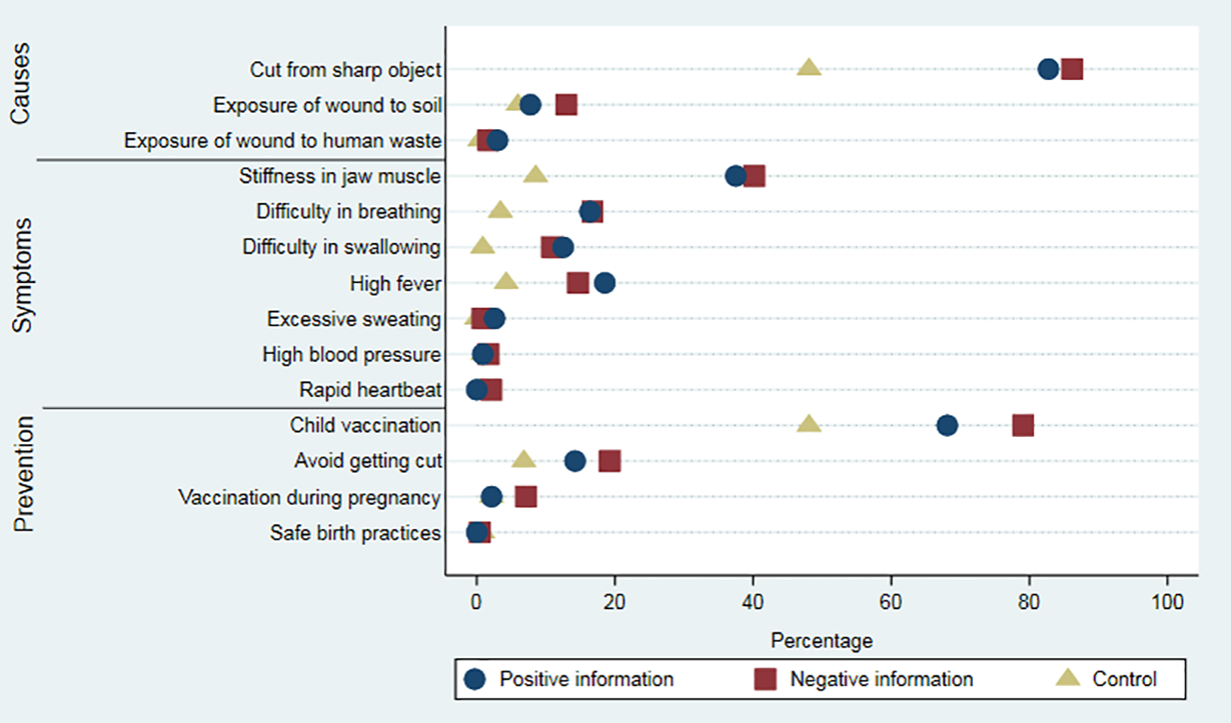
Tetanus knowledge items by treatment group. The figure shows the treatment effect on each knowledge item. Each point is the mean of the knowledge item at endline.

Fig 2 shows the effect of the information intervention on each knowledge indicator, showing that the negative framing had a larger impact than the positive framing on most measures of knowledge (differences were significant at the 5% level for knowledge of rapid heartbeat as a symptom of tetanus, and knowledge of child vaccination and vaccination during pregnancy as methods of prevention).

**Fig 3:**
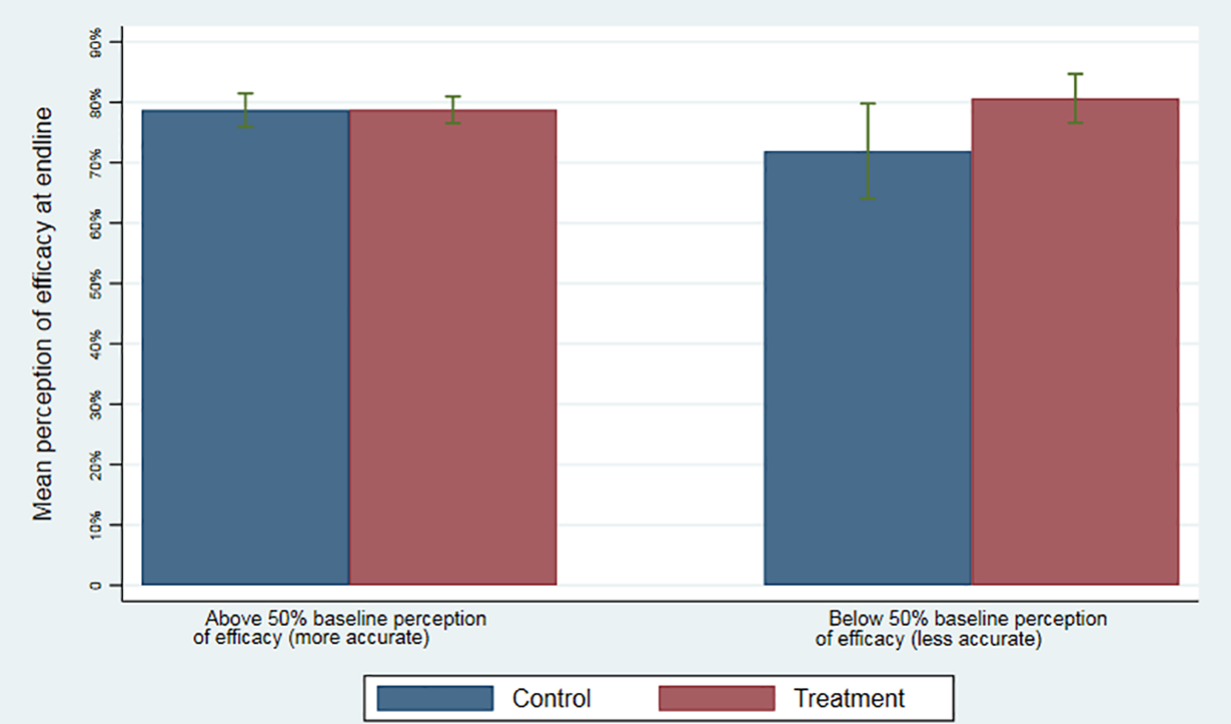
Effect of information on perceptions of tetanus vaccination efficacy for women with different levels of baseline perceptions. The figure shows the effect of the information intervention on perceptions of vaccine efficacy in 2 subgroups of mothers: those with initial perceptions of efficacy of the tetanus vaccine above 50% and those with initial perceptions of efficacy below 50%. Error bars indicate 95% CIs.

We conducted several additional analyses to better understand the main findings. First, Fig 3 shows the effect of the information intervention on perceptions of vaccine efficacy for 2 subgroups of mothers with perceptions of efficacy at baseline above and below 50%. The intervention had a significant positive effect on mothers who, at baseline, believed the DPT vaccine to have an efficacy level below 50% (difference: 0.88, 95% CI: 0.06 to 1.69, *p =* 0.04). The intervention did not have an effect on mothers who were already convinced of the efficacy of the DPT vaccine—i.e., those who had perceptions of efficacy above 50% at baseline (difference: 0.01, 95% CI: −0.36 to 0.37, *p =* 0.97). Second, using variation in the geographical density of study households assigned to the information intervention, we found no evidence of spillovers. Proximity to study households that received the information intervention did not affect the probability of children receiving DPT3 (S4 Table).

The total cost of the information intervention was $11,353, of which $2,923 (26%) was start-up costs and $8,430 (74%) was implementation costs. Table 4 shows that the cost of the information intervention was $165 per additional child with DPT3, $109 per additional child with measles vaccine, $186 per DALY averted, and $5,572 per under-5 death averted. The cost per DALY averted was considerably lower than standard thresholds used to define highly cost-effective interventions. The approach of the World Health Organization is to use a threshold based on the country’s gross domestic product per capita ($793 in the case of Uttar Pradesh), while the World Bank’s World Development Report 1993 recommended at the time $150 per DALY ($246 in current dollars). Under our scale-up scenario, the cost of the information intervention falls to $95 per DALY averted and $2,840 per under-5 death averted. While these estimates are only for the purposes of illustration, they encompass a wide range of valuations for which the intervention would be considered highly cost-effective.

**Table 4:** Cost-effectiveness of the information intervention.

Outcome	Costing scenario	
	Actual	NGO scale-up
Cost per mother given information	$24.10	$12.28
Cost per additional child vaccinated with DPT3	$165.10	$84.13
Cost per additional child vaccinated for measles	$109.57	$55.83
Cost per DALY averted	$185.57	$94.56
Cost per under-5 death averted	$5,572.63	$2,840.02

Estimates exclude survey and research costs. For details of the assumptions underpinning the NGO scale-up scenario, see S4 Text.

DALY, disability-adjusted life year; DPT3, 3 doses of diphtheria–pertussis–tetanus vaccine; NGO, non-governmental organisation.

## Discussion

This paper presented evidence on the role of information in raising demand for immunisation in India. Our analysis yielded 3 key findings. First, providing mothers of unvaccinated or incompletely vaccinated children with information on tetanus and the benefits of vaccination substantially increased immunisation coverage of DPT3, full immunisation, and measles. The large effect on measles vaccination was not anticipated, given that the information intervention focused solely on tetanus. We speculate that the increase in measles vaccination was generated by increased engagement with the public health system and, in turn, health workers ensuring children were up to date on all their vaccines, not just DPT3. Second, the framing of the information did not appear to generate large differences in outcomes. Although the effects of negative framing were consistently larger than when information was framed as a gain, differences between the 2 groups were small and rarely significant. Third, information improved mothers’ knowledge of causes of, symptoms of, and methods of prevention against tetanus. There was no effect on perceptions of vaccine efficacy, but there was suggestive evidence of an increase in perceptions of efficacy for mothers who initially had inaccurate perceptions.

The findings leave open the possibility that the intervention worked through various channels, most notably by increasing basic awareness on the existence of the DPT vaccine as a method of prevention. An alternative explanation is that the information served simply to remind mothers to get their child vaccinated, in which case cheaper interventions—such as SMS text message reminders—could be envisaged. We caution against such an interpretation. Not only do our results on knowledge suggest otherwise, but also all study participants were exposed to questions about vaccines in the baseline survey, making the immunisation status of the child salient. We also note that studies of the effect of text message reminders on vaccination uptake in developing countries have produced mixed results, showing either small or zero effects [24–28]. The effectiveness of reminders relative to the information intervention we tested should be the focus of future research.

### Strengths and limitations

A key strength of the study is that it is one of the first to evaluate the effectiveness of information provision on immunisation coverage in India, where a substantial proportion of the world’s unvaccinated children live. Other strengths of the study include the randomised design, the wide range of outcomes measured, and the novel nature of the information intervention. The information intervention targeted the mothers of unvaccinated/incompletely vaccinated children rather than the wider population of mothers. To inform the optimal design of the intervention, we tested 2 variants of how to frame the information.

The study had a number of limitations. We conducted an individual rather than a cluster randomised controlled trial, making contamination a potential concern. However, the information was given in private, and our analysis suggests that the outcomes of neighbours were not influenced by their proximity to treated households. If contamination was present, our results would provide an underestimate of the effect of the information intervention. We did not achieve the target sample size for the treatment group analysis such that the study was underpowered to detect small differences between the 2 intervention groups. Further research on framing in the context of immunisation is warranted to optimise the intervention. Qualitative research may have shed more conclusive light on how the intervention worked. We were unable to locate more than 400 potentially eligible women who could not be found at home. This may have limited the extent to which our study sample was representative of the wider population. Finally, the follow-up period was only 7 months. Tracking the study participants for longer would have provided evidence on whether the effects on outcomes were sustained.

### Interpretation in light of other studies

Our study contributes to the growing body of evidence on interventions to increase uptake of vaccinations. A recent systematic review and meta-analysis found that demand-side interventions lead to an increase in the uptake of vaccinations, with a relative risk of 1.30 (95% CI: 1.17 to 1.44) [8]. The relative risk in this study was 1.52 (95% CI: 1.21 to 1.91), making this information intervention among the more effective ways to increase demand. The effect of information has been studied in the context of other health behaviours—such as maternity care [29], water purification [30–33], prevention of worms [34], malaria bed net use [35], and circumcision for HIV prevention [36]—with mixed results. We also contribute to evidence on the framing of health information. Our findings support a review that concluded, contrary to expectations, that there is no consistent effect of framing on health behaviours [15].

### Generalisability

For policymakers, the findings suggest that targeted information may be a highly cost-effective means of increasing uptake of childhood immunisation. While caution must be taken in generalising the findings beyond the study setting, we highlight a number of context-specific factors that may be relevant when considering the wider relevance of the findings. First, the 6 study districts were not the worst performing in the state. In areas where immunisation rates are lower, such as remote rural areas in Uttar Pradesh and other Indian states, the intervention might produce larger effects as long as the vaccine supply is in place. Second, a local organisation implemented the intervention; however, any future scale-up would likely rely on government delivery channels, which may reduce costs but at the same time could present risks in terms of implementation fidelity. Third, we note that mothers’ knowledge and perceptions of efficacy at baseline were quite low; the intervention is likely to be less effective in areas where awareness and knowledge levels are higher. Finally, the supply of vaccines must be in place, at least intermittently, if information is to have any effect in increasing immunisation rates.

In light of the results on framing, a prudent strategy would be to adopt the negative framing of the information script since there are no cost implications. There may of course be cheaper ways of delivering the same information to parents. In the context of India, one obvious option could be to engage ASHAs given that they are integral to the delivery of community health services and have a strong focus on maternal and child health. A potential concern would be the loss of fidelity in the implementation of the intervention. At the same time, the messages are simple and quick to deliver.

Policymakers may also want to consider whether information should be combined with other interventions. Recognising the multiple barriers to behaviour change that exist, a small number of studies have combined information with price subsidies or monetary incentives. Ashraf et al. [37] studied the effects of door-to-door marketing of water purification products with different combinations of price subsidies and information about the benefits of the target product compared to a traditionally used product. They found that additional information increased the effectiveness of price subsidies by 60%. Banerjee et al. [38] considered demand- and supply-side interventions in combination, showing that incentives alongside immunisation camps were far more effective in raising immunisation rates than increasing supply alone.

There are a range of unanswered questions about how best to deliver information to improve immunisation coverage. Alternative, less costly ways of delivering information could make use of social networks. There is a growing literature on social networks being exploited to spread information and behaviours, e.g., by targeting highly connected individuals, nominated friends of individuals, or community leaders [39,40]. Understanding how information spreads is an active area of research that could yield useful insights for public health [41].

## Conclusion

Our results demonstrate that targeted and clear information delivered to mothers of unvaccinated/incompletely vaccinated children can be effective in improving immunisation coverage. These findings contribute to a growing body of evidence on what are the most effective strategies to improve vaccination rates in developing countries. Although the barriers to immunisation uptake are multiple, ranging from social norms to the reliability of supply systems, in contexts where knowledge and awareness are a key binding constraint, interventions that provide information to parents and carers of unvaccinated children have the potential to be a simple and cost-effective way of increasing demand for immunisation.

## Supporting information

S1 CONSORT checklist (DOCX)

S1 Dataset (XLS)

S1 Fig Comparison between selected, study district, and state clusters for key census indicators. (TIF)

S2 Fig Information leaflet: Information framed as a loss. The images have been deleted for copyright reasons.
(TIF)

S3 Fig Information leaflet: Information framed as a gain. The images have been deleted for copyright reasons.
(TIF)

S1 Protocol (DOCX)

S1 Table Characteristics of study participants surveyed 9 months prior to start of study. Values are *n/N* (%) for binary outcomes and mean (standard deviation) for continuous outcomes. None of the differences between groups are significant at the 5% level. Scheduled caste and scheduled tribe are among the most disadvantaged socioeconomic groups in India. Other backward caste is an additional category of socially and economically disadvantaged people. All 3 are official terms used by the government of India and given explicit recognition in India’s constitution.
(DOCX)

S2 Table Adjusted estimates, pooled analysis, and treatment group analysis. All regressions are ordinary least squares, adjusted for child age and baseline measure of the outcome. Treatment effects are in terms of absolute differences. Results from the 2 analyses are presented: pooled analysis and treatment group analysis.
(DOCX)

S3 Table Treatment group analysis with a Bonferroni correction. (DOCX)

S4 Table Spillover effects. All regressions are ordinary least squares. The 2 information groups are pooled together as a single treatment group. Standard errors are presented in parentheses. The share of treated neighbours is the number of other study participants who were assigned to receive the information intervention divided by total number of study participants within a specified radius. * *p <* 0.10, ** *p <* 0.05, *** *p <* 0.01.
(DOCX)

S5 Table Study setting: Demographic and health indicators. Sources are the Indian Census 2011 and the Annual Health Survey 2012–13. The maternal mortality ratio estimates apply to groups of districts within the state due to sample size limitations.
(DOCX)

S1 Text Study setting. (DOCX)

S2 Text Sampling strategy. (DOCX)

S3 Text Method to measure perceived efficacy of DPT vaccine. (DOCX)

S4 Text Cost-effectiveness calculations. (DOCX)

## Abbreviations

ASHA: accredited social health activist
BCG: Bacillus Calmette–Guérin
DALY: disability-adjusted life year
DPT: diphtheria–pertussis–tetanus
DPT3: 3 doses of diphtheria–pertussis–tetanus vaccine
LMICs: low- and middle-income countries
