# Supplementary figures and images for: Effect and cost-effectiveness of educating mothers about childhood DPT vaccination on immunisation uptake, knowledge, and perceptions in Uttar Pradesh, India: A randomised controlled trial
Source: PLoS Med. 2018 Mar 6;15(3):e1002519. doi: 10.1371/journal.pmed.1002519 (PMC5839535; doi:10.1371/journal.pmed.1002519)

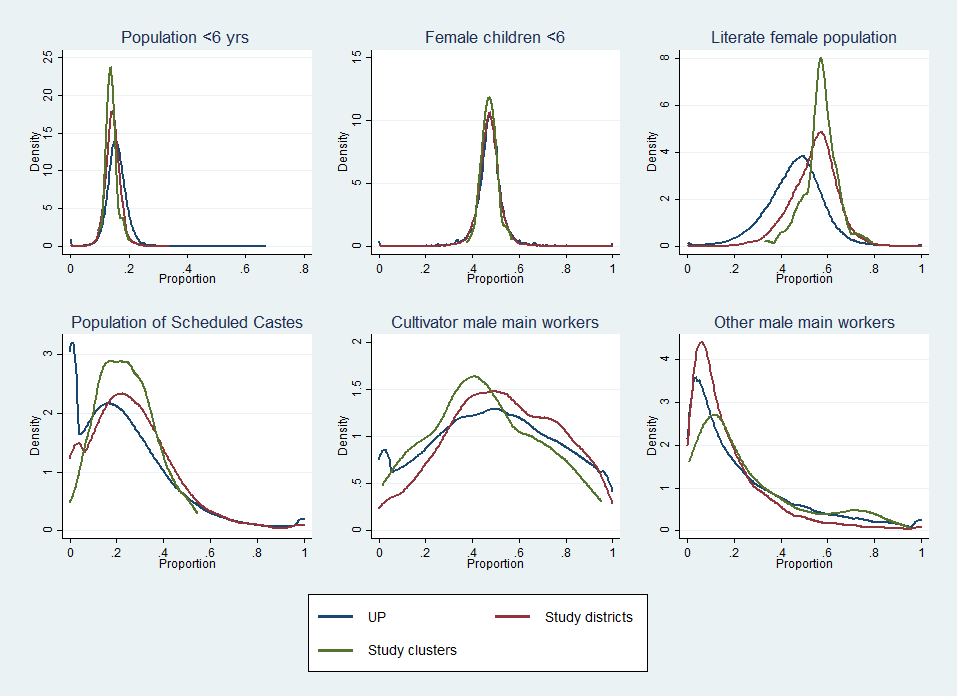

Supplement: S1 Fig — (TIF) [file pmed.1002519.s003.tif]

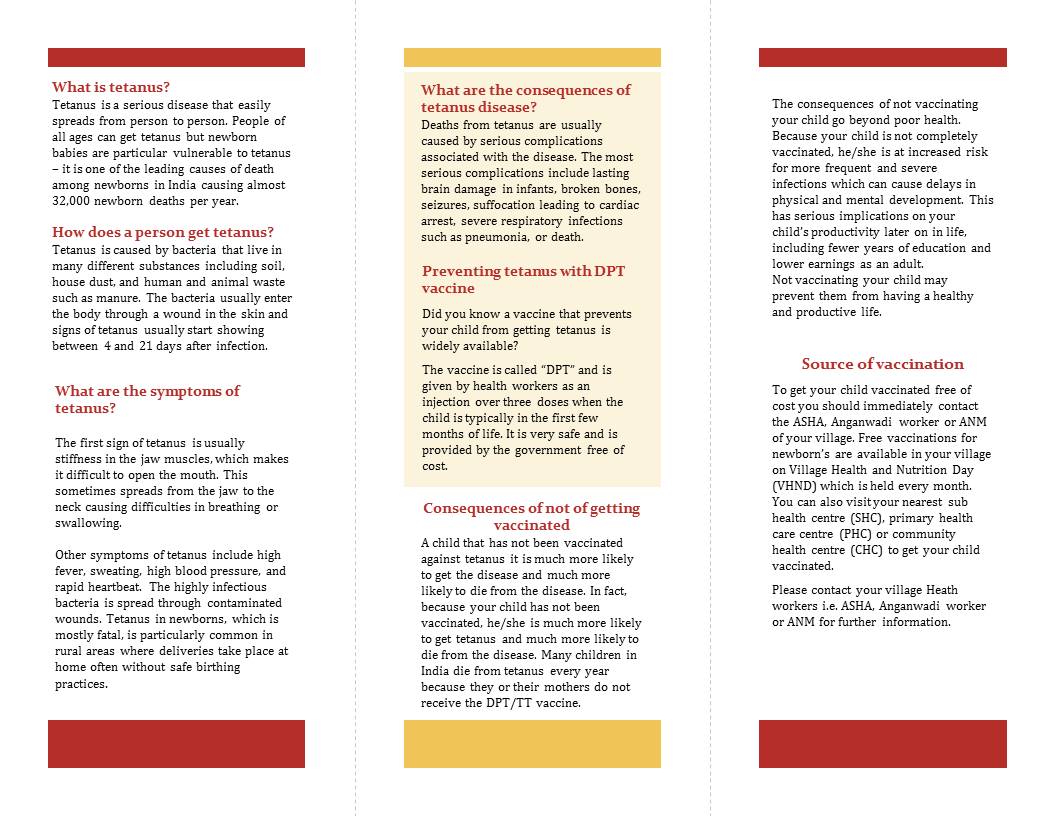

Supplement: S2 Fig — The images have been deleted for copyright reasons. (TIF) [file pmed.1002519.s004.tif]

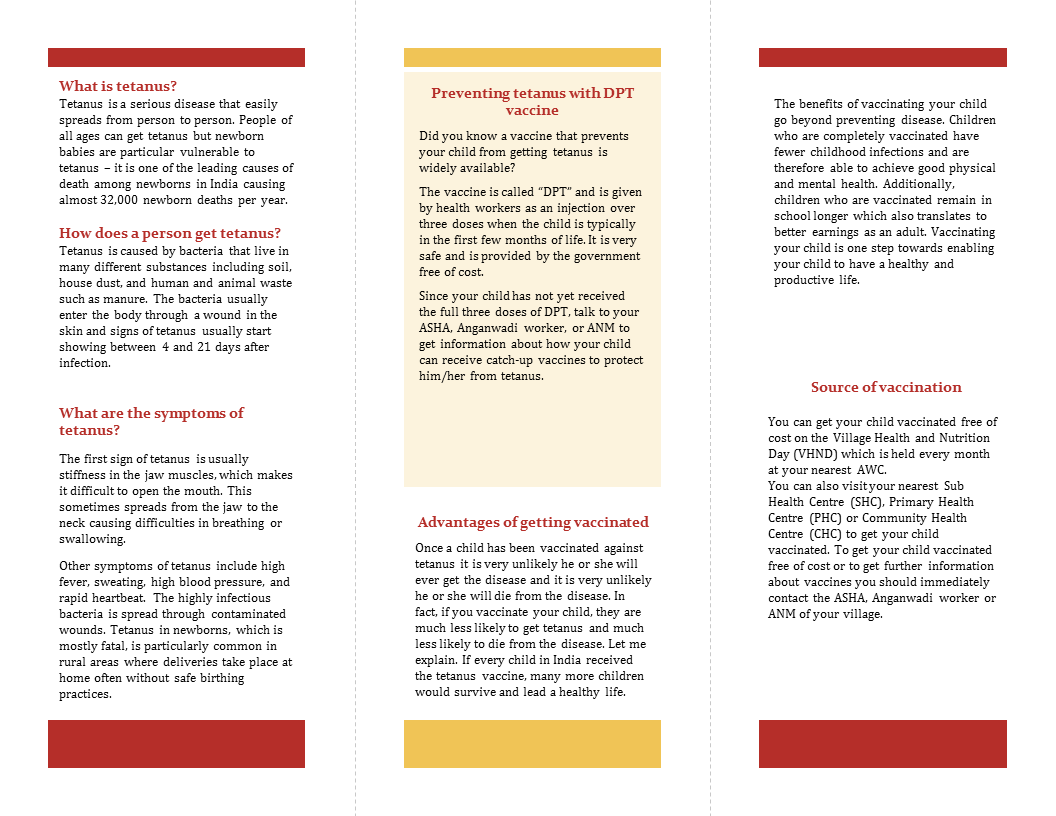

Supplement: S3 Fig — The images have been deleted for copyright reasons. (TIF) [file pmed.1002519.s005.tif]
