# Supplementary material for: Effect and cost-effectiveness of educating mothers about childhood DPT vaccination on immunisation uptake, knowledge, and perceptions in Uttar Pradesh, India: A randomised controlled trial
Source: PLoS Med. 2018 Mar 6;15(3):e1002519. doi: 10.1371/journal.pmed.1002519 (PMC5839535; doi:10.1371/journal.pmed.1002519)
Supplement: S1 Protocol — (DOCX) [file pmed.1002519.s006.docx]

# Text S6. Study protocol

Information and the demand for preventative healthcare: a randomised controlled trial of improving household perceptions on the efficacy of DPT immunisation in Uttar Pradesh, India

Principal Investigator:

Timothy Powell-Jackson (LSHTM)


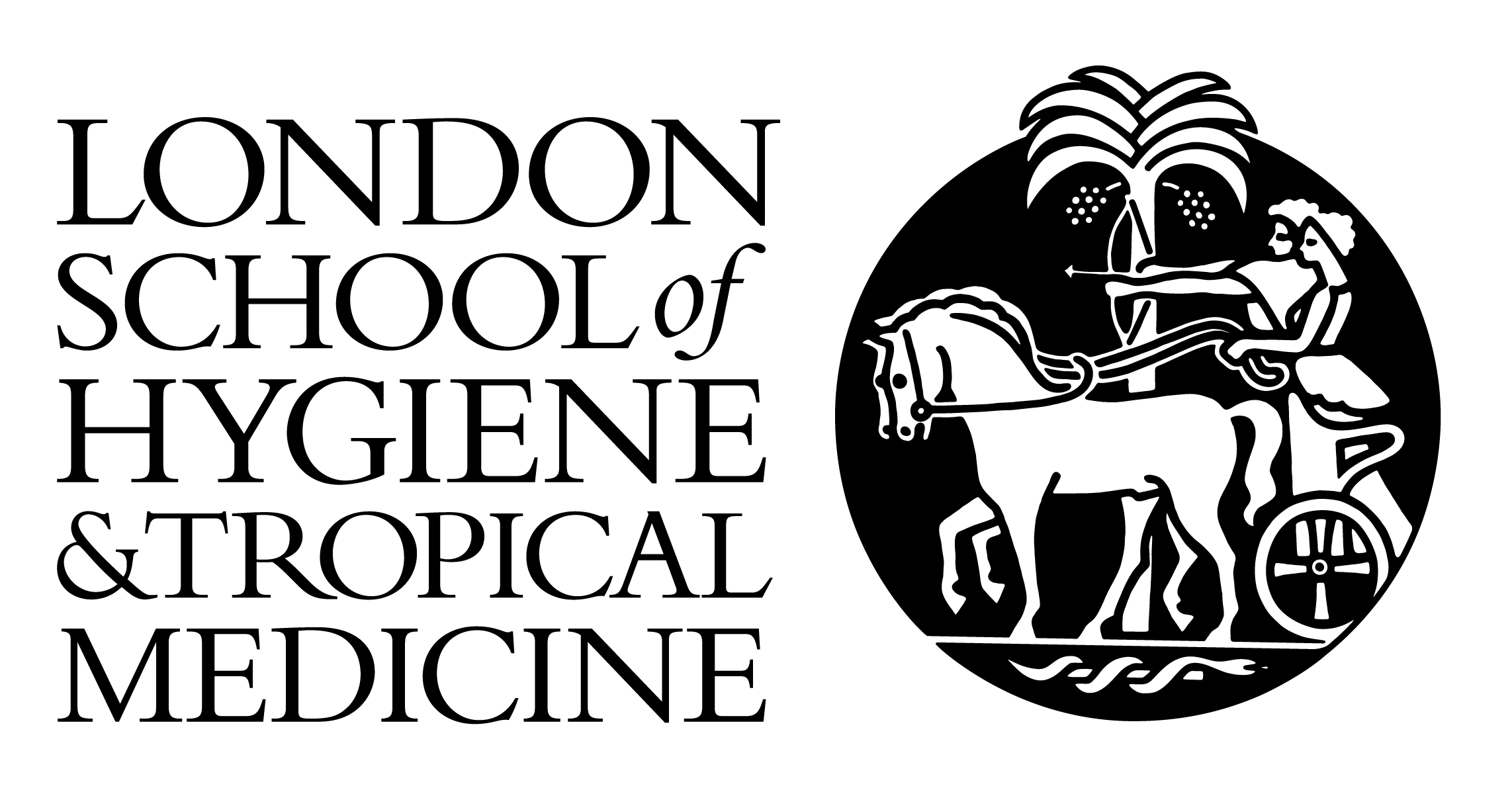


# Background

## Information and the Demand for Healthcare

There is now a strong body of evidence of what interventions are most effective in improving health in low- and middle-income countries ([Jamison, 2006](#_ENREF_9)). This is particularly true in the case of child health ([Jones et al., 2003](#_ENREF_10)). Policymakers have available to them a range of interventions that have been shown to be not only effective but also cheap to deliver. Indeed such interventions have contributed to remarkable improvements in child mortality in many developing countries ([Liu et al., 2014](#_ENREF_13)). But there remain important gaps in the coverage of interventions both between and within countries ([Bhutta et al., 2010](#_ENREF_3), [Victora et al., 2012](#_ENREF_30)) and little is known about how to ensure widespread coverage in the population ([Travis et al., 2004](#_ENREF_28)) despite calls for greater focus on implementation research ([Sanders and Haines, 2006](#_ENREF_24)).

Lack of information is often cited as a primary cause of low investment in preventative health products and services. As a result, provision of health information is commonly used to try to increase demand for health products and services in developing countries. Providing information is thought to impact the overall demand for health products and services, as well as the elasticity of demand, by allowing consumers to purchase more appropriate products or by changing their perception of quality ([Ashraf et al., 2013](#_ENREF_1)). Information interventions also have the benefit of being low cost to implement, and their effects are thought to be sustainable if they succeed in changing behaviours ([Takasaki and Sato, 2013](#_ENREF_26)). For health interventions such as vaccinations, even short-lived changes in behaviour could lead to large health impacts because once vaccinated the child is immunised for years to come.

Though a large literature on the impact of information provision on health behaviours exists in developed countries, much less is known of the effects of health information in developing countries ([Tarozzi et al., 2010](#_ENREF_27)). Few studies succeed in isolating the impact of health information alone on health behaviours, with many studies bundling health education together with other interventions, or failing to include a control group ([Luo et al., 2012](#_ENREF_15)). The evidence overall is decidedly mixed, with the effectiveness of such interventions varying with the nature of the intervention, such as the form and specificity of the information provided, the intensiveness of the intervention and the removal of additional barriers.

The study will test the extent to which information designed to improve household perceptions of the efficacy of healthcare increases service uptake. Because it would be unethical to attempt to provide households with information on services for which there is no rigorous evidence on efficacy, the general principle will be tested using the example of DPT vaccine, for which there is universally accepted evidence on efficacy. The study takes place in Uttar Pradesh where a substantial proportion of children are not fully immunised despite the availability of services.

## Literature Review

This section reviews the evidence on health information interventions in developing countries. Table A1 in Appendix 1 summarises the main studies that have been undertaken on the effect of information interventions on health-related behaviours. The evidence on the overall effectiveness of information provision for changing behaviour, and therefore uptake of health services, is mixed and fairly limited. Effects vary with the nature of the intervention, and more research is needed to ascertain the conditions under which health information campaigns might be more effective and the precise mechanisms through which they work.

A systematic review has been conducted on the effect of informing populations about the safety of their drinking water supply ([Lucas et al., 2011](#_ENREF_14)). The authors point to the limited nature of available evidence due to a lack of studies with robust control or comparison groups, and state that it is not yet possible to draw firm conclusions of their effect. The authors also state the importance of context, and the need to consider the format of intervention provided and the methods of dissemination. Informing households on the safety of their water supply is one of the more common forms of information interventions, and has been found to be effective ([Jalan and Somanathan, 2008](#_ENREF_8), [Madajewicz et al., 2007](#_ENREF_17)) due to the individually targeted nature of the information provided. Jalan et al (2008) found that households in India informed that their drinking water tested positive for faecal contamination were 11% more likely to begin some form of home purification in the next eight weeks, and spent $7.24 (at PPP) more on purification, than households that received no information. A study by Madajewicz et al (2007) in Bangladesh found that being informed of an unsafe concentration of arsenic in a household’s well raised the probability of the household changing to another well within one year by 37%. Similarly, Luoto et al (2011) find that sharing information on whether community water sources are contaminated increases use of water purification devices by 7-10%, though additional information of the individual household’s own water quality has no further effect. By contrast, more generalised information on, for example, the dangers of worm infection and the importance of wearing shoes to prevent hookworm, had no impact on the purchase of shoes ([Meredith et al., 2013](#_ENREF_18)).

The format of information provided is also important in terms of whether it is framed positively or negatively. There is disagreement in the literature, with some suggesting that a positive frame works best to change behaviour, and others stating that due to loss aversion, gains relative to the status quo are often valued less than avoiding losses relative to the status quo ([Luoto et al., 2011](#_ENREF_16)). Interventions can also use a contrast frame that first describes a problem, and then presents a solution; Luoto et al (2011) found that messages that framed safe water technologies as both avoiding disease and improving health, as opposed to improving health alone, raised usage rates by 4-6%. In terms of the format of information, variations in perceptions of risk are also important to consider. A study comparing ‘continuous’ communication on health risk (marking wells with the level of arsenic-contamination) compared with binary marking of wells as good or bad found that continuous communication in fact decreased the impact of arsenic level on the probability of switching to a new source of water ([Tarozzi et al., 2010](#_ENREF_27)). Additionally, a review of different approaches to providing health risk information found that numerical information about the probability of health risks occurring is less understandable and effective than information about the causes and consequences of a health problem, and has less success in systematically influencing perceptions of health risk ([Rothman and Kiviniemi, 1999](#_ENREF_23)). Takasaki and Sato (2013) note the variation in the effectiveness of their information intervention based on cognitive ability; information provision decreased uptake of ANC among illiterate women, suggesting that ill-processed information can have negative effects. Other key social factors identified as determining the likelihood of behaviour change include gender, poverty, stigma, convenience and local social structures ([Lucas et al., 2011](#_ENREF_14))

The intensity of informational interventions has also been raised as a potential determinant of their effectiveness; Cairncross et al (2005) assess the effects of an intensive educational programme on hygiene, including group meetings, exhibitions, health camps, films, street drama, health clubs, classes and medical camps conducted over many months, which had positive effects on hygiene knowledge and practice several years after the intervention had ended. The economics literature has largely emphasised that behaviour change occurs slowly with the accumulation of information, ([Ashraf et al., 2013](#_ENREF_1)), and one off door-to-door visits may therefore be less likely to have an effect ([Meredith et al., 2013](#_ENREF_18)).

The outcomes under study in informational interventions can be a change in knowledge, behaviour, or overall health. A change in knowledge may be more likely than a change in behaviour; while 98% of people could correctly state whether their well was safe following information provision in Araihazar, Bangladesh, knowing that a well had unsafe concentration of arsenic only raised the probability of changing water supply by 37% ([Madajewicz et al., 2007](#_ENREF_17)). In India, though provision of information about nutrition improved the theoretical knowledge of mothers, it did not results in an increase in infant weight ([Singh, 2011](#_ENREF_25)). This is not suprising; even if information increases the perceived value of an item or behaviour, the costs of purchasing items or changing behaviour may be too high; households that did change their water supply to a different well in the Madajewicz study faced a fifteen fold increase in time spent obtaining water. Meredith et al (2013) find that while information substantially increased knowledge about worms, this did not translate into an increase in demand, while Ashraf et al (2013) find that information does not affect the level of investment made in health products, but does affect the slope of the demand curve.

Other studies ([Godlonton et al., 2011](#_ENREF_7), [Rhee et al., 2005](#_ENREF_22), [Givord and Romanello, 2011](#_ENREF_6)) have found that information can increase the demand for health care. Opportunistic provision of information about assisted childbirth through a local voluntary worker during a tetanus vaccination campaign increased the level of demand for maternal care and assisted delivery in Mali ([Givord and Romanello, 2011](#_ENREF_6)), while a study by Rhee et al (2005), also in Mali, found that provision of information about health risks increased the likelihood of households paying to have their bed nets impregnated with insecticides when offered the service by a village worker. In Malawi, Godlonton et al (2011) randomly disseminated information on circumcision and HIV risk to households following a questionnaire, and found a significantly increased likelihood of buying condoms and decreased risky sexual behaviour in uncircumcised men after one year. However, even when behaviour change is documented, the ultimate impact on health can be questionable; Luo et al (2012) carried out face-to-face education sessions and dissemination of written materials for parents at their children’s schools with the aim of reducing iron-deficiency anaemia among primary school students in rural China, and found little evidence of changes in blood haemoglobin concentration or anaemia status.

Recognising the multiple barriers to behaviour change that exist aside from lack of information, informational interventions are commonly combined with the removal of additional barriers to health products or services, such as combining information provision with price subsidies ([Ashraf et al., 2013](#_ENREF_1)) or conditional cash transfers ([Takasaki and Sato, 2013](#_ENREF_26)). These studies have varying results: Ashraf et al study the effects of door-to-door marketing of water purification products with varying combinations of price subsidies and information about the benefits of the target product compared to a traditionally used product. They find that additional information increased the effectiveness of price subsidies by 60%. However this reflects a shift in consumers from the familiar product to the target product, and not an overall increase in demand for water purification. In contrast, Tagasaki and Sato (2013) compare the effects of providing basic information about the benefits of antenatal care with conditional cash transfers, or a combination of the two interventions, and find that cash transfers increase rapid uptake of ANC services, but use of services is not repeated, while information has no additional impact on uptake of services. Unlike the Ashraf (2013) and Rhee (2005) studies, this intervention required additional action beyond the time of the information being provided, which may partly explain the lack of effect. Singh et al (2011) combine information provision to mothers in the form of a book of government recipes for young children with performance-related pay for child care workers, and find that the combination of interventions reduces weight for age malnutrition by 4.2% in 3 months, but that the individual intervention effects are negligible.

Where informational interventions do succeed in changing behaviours, their effects are thought to be sustainable, and this is supported to some extent by the evidence. Godlonton et al (2011) find that providing information on circumcision and HIV risk decrease risky sexual behaviour one year after the intervention, suggesting lasting effects on knowledge and behaviour. Additionally, the assessment of a hygiene promotion intervention in Kerala nine years after its implementation was recalled by 74% of study households. Health education classes were recalled by 83% of households, and recall was positively associated with improved hygiene knowledge and practice. However, the authors acknowledge that many such campaigns take place in Kerala, and some respondents may have been referring to a different campaign ([Cairncross et al., 2005](#_ENREF_4)).

## Link with Other Research Activities

The research detailed in this study protocol is embedded within a larger research project to evaluate the impact of a social franchising in Uttar Pradesh. The social franchise model is funded by MSD for Mothers and implemented by two NGOs – Pathfinder International (lead) and World Health Partners (partner). They work in three districts of Uttar Pradesh – Kannauj, Kanpur Nagar and Kanpur Dehat. The social franchise project aims to increase access to and use of basic obstetric care, emergency obstetric care and family planning services. It works towards three objectives over its three year lifetime to:1) establish the “Sky social franchise” network of private health providers and functional referral centres offering affordable antenatal care, emergency obstetric care, and family planning services; 2) strengthen capacity of and linkages between rural private and public sector health providers to offer high quality services; and 3) improve community awareness, demand and linkages with maternal health services among rural populations.

Examining whether information can increase demand for and uptake of healthcare will indirectly contribute to our understanding of one of the ways in which social franchising is anticipated to work. Branding and advertising are an important part of social franchising that is theorised to improve household care seeking practices ([Montagu, 2002](#_ENREF_21)). They provide a signal of quality. If viewed as credible, such a signal can be expected to raise perceptions of quality and under certain conditions increase demand for healthcare at social franchisees. Proponents argue that social franchising has the potential to transform the private health sector in developing countries. Yet this general principle or mechanism that underpins the model remains largely untested.

The evaluation of the social franchise project is itself part of a larger programme of work to evaluate the MSD for Mothers Initiative, being undertaken by a team of researchers at the London School of Hygiene and Tropical Medicine. The 10-year, $500 million initiative launched in September 2011 aims to reduce maternal mortality around the world. The initiative focuses on averting deaths from postpartum haemorrhage (PPH) and hypertensive disorders of pregnancy (HDP), and on preventing unintended pregnancy via family planning (FP). The MSD for Mothers’ strategy is currently being implemented via a three-pillar program of projects aimed at: 1) product innovation; 2) accelerating access to essential maternal health care & family planning supplies & services; and 3) awareness and advocacy at global and national level.

# Study Setting and Intervention

## Uttar Pradesh

Uttar Pradesh is India’s fifth largest and most populous state with approximately 199.8 million people living in 18 divisions and 75 districts. If Uttar Pradesh were a country, it would be the fifth largest in the world. The proportion of the population living in rural areas is 77%, and this contributes to the largest share of India’s rural population (18.6% in 2011). Over half of the population is literate and 31% live below the poverty line. In 2010-2011, Uttar Pradesh’s total fertility rate was an estimated 3.6, and the median age at first live birth for women between 15-49 years is 22 years. Maternal and infant mortality remain high, with most recent estimates of the MMR at 345 deaths per 100,000 live births and infant mortality at 71 deaths, in the first year of life, per 1000 live births (Table 1).

The study is located in six districts of Uttar Pradesh: Kannuaj, Kanpur Nagar, Kanpur Dehat, Auraiya, Etawah, and Fatehpur. These districts vary only modestly in terms of demographic and health indicators, except for one clear outlier (Table 1). Kanpur Nagar is predominantly urban, with higher literacy and lower mortality than the state average. By contrast, the other districts are more typical of the state as a whole. Largely rural, they have poor literacy and high rates of maternal and child mortality that are comparable with the less developed countries in the world.

Table 1. Demographic and health indicators

| Indicator | Uttar Pradesh | Kannauj | Kanpur Nagar | Kanpur Dehat | Auraiya | Etawah | Fatehpur |
| --- | --- | --- | --- | --- | --- | --- | --- |
| Population (in millions) | 199.8 | 1.7 | 4.6 | 1.8 | 1.4 | 1.6 | 2.6 |
| Rural population (%) | 78 | 83 | 34 | 90 | 83 | 77 | 88 |
| Literacy (%) | 57 | 61 | 71 | 65 | 67 | 67 | 57 |
| Scheduled caste (%) | 21 | 19 | 18 | 26 | 28 | 25 | 25 |
| Scheduled tribe (%) | 0.57 | < 0.1 | < 0.1 | < 0.1 | < 0.1 | < 0.1 | < 0.1 |
| Fertility (lifetime) | 3.3 | 3.3 | 2.1 | 2.8 | 3.5 | 3.1 | 3.5 |
| Maternal mortality ratio (per 100,000) | 258 | 240 | 240 | 240 | 240 | 240 | 283 |
| Under five mortality (per 1,000) | 90 | 102 | 50 | 94 | 84 | 85 | 81 |
| Infant mortality (per 1,000) | 68 | 79 | 37 | 65 | 58 | 56 | 55 |
| Neonatal mortality (per 1,000) | 49 | 55 | 24 | 41 | 41 | 41 | 39 |
| Notes: Sources are the Census 2011 and the Annual Health Survey 2012-13. The MMR estimates apply to groups of districts within the state due to sample size limitations. | | | | | | | |

A substantial proportion of children do not receive the vaccines recommended by the Government of India (Table 2). Just under two-thirds of children receive three doses of DPT in the state. Coverage of DPT3 in the study districts is either around the state average or above, with modest variation between districts. Coverage of measles largely mirrors that of DPT3. A higher proportion of children receive the BCG vaccine, the first of the routine immunisations given at birth. Only half of all children in the state are fully immunised against the six main vaccine preventable diseases implying that there are important barriers to uptake.

Table 2. Immunisation coverage for children aged 12-23 months

| Indicator | Uttar Pradesh | Kannauj | Kanpur Nagar | Kanpur Dehat | Auraiya | Etawah | Fatehpur |
| --- | --- | --- | --- | --- | --- | --- | --- |
| Children 12-23 months received 3 doses of DPT | 63.2 | 59.1 | 72.4 | 72.4 | 59.5 | 69.2 | 65.5 |
| Children 12-23 months received measles vaccine | 65.8 | 61.2 | 75.1 | 79.7 | 60.2 | 75.7 | 70.7 |
| Children 12-23 months with immunization card | 71.7 | 66.0 | 92.4 | 92.0 | 80.3 | 92.1 | 88.2 |
| Children 12-23 months received BCG vaccine | 86.3 | 90.5 | 89.4 | 92.6 | 84.9 | 91.3 | 91.1 |
| Children 12-23 months received 3 doses of polio | 64.1 | 59.1 | 73.2 | 70.8 | 60.2 | 72.7 | 65.5 |
| Children 12-23 months fully immunized | 52.7 | 46.4 | 59.2 | 61.9 | 46.2 | 58.0 | 51.5 |
| Notes: Source is the Annual Health Survey 2012-13. | | | | | | | |

## Immunisation Schedule in India

The Indian Academy of Paediatrics provides information on the recommended immunisation schedule for children aged 0 to 18 years. It is recommended that three doses of DPT are given to children at 6 weeks, 10 weeks and 14 weeks. The minimum age for this vaccine is 6 weeks. If any of these are missed, then the recommended age for catch-up is any time until the child is 12 months. While two DPT boosters are recommended at 15-18 months (fourth dose) and 4-6 years (fifth dose) respectively, the standard immunisation indicator for this vaccine is defined by whether a child aged 12-23 months has received three doses of DPT.

## Information Intervention

### Description of intervention

The study will test an intervention that provides mothers with information concerning the DPT vaccine. The information script will focus primarily on tetanus given that it has the greatest disease burden of the three diseases ([Million Death Study et al., 2010](#_ENREF_20)). Specifically, it will describe the symptoms of tetanus, possible health consequences, the individual benefit of the combination vaccine in terms of mortality and morbidity gains, wider community benefits associated with herd immunity, and where the family can get the child vaccinated at what cost and up until what age.

There will be two versions of the script. The first will frames the information on DPT vaccination as gains – eg. X% less likely to get tetanus, Y% more likely to live if the child is vaccinated and Z number of fewer tetanus deaths per year if every child was vaccinated in India. The second will frame information on DPT vaccination as losses – eg. X% more likely to get tetanus, Y% more likely to die if the child is not vaccinated and Z number of deaths per year because not every child is vaccinated in India. The information script will be delivered to the mother in the privacy of her own home. Visual aids will also be used to help provide the information in an accessible manner. There will a short question and answer session to ensure comprehension of the information.

### Anticipated effects

A health information intervention could theoretically have one of three effects. If individuals lack accurate information and their priors are lower than the true efficacy of healthcare, then giving information could motivate households to increase uptake of healthcare. On other hand, if individuals hold perceptions that are more optimistic than the true efficacy of healthcare, providing information could lead to reduced demand. Finally, providing information will likely have no effect if families already hold accurate beliefs about the efficacy of healthcare, heavily discount the future, or face supply-side constraints that make it difficult to increase uptake of healthcare. Peer effects – the effect on families socially connected to recipients of the intervention – can be expected primarily through further dissemination of the information between families although other channels are also possible ([Dupas, 2014](#_ENREF_5), [Kremer and Miguel, 2007](#_ENREF_12)).

How decisions are framed can have a powerful influence on choices. In particular, people tend to be sensitive to the gains and losses associated with the outcome of a decision because of loss aversion. This refers to people’s tendency to prefer avoiding losses to acquiring gains. A large literature on reference dependent preferences demonstrates behaviour consistent with a notion of loss aversion ([Kahneman and Tversky, 1979](#_ENREF_11), [Tversky and Kahneman, 1991](#_ENREF_29)). Lab experiments have consistently demonstrated that subjects are more responsive to protocols framed as losses than to protocols framed as gains and there is a growing body of field experiments testing the effect of the loss frame in marketing messages on product demand ([Bertrand et al., 2010](#_ENREF_2)). In light of the literature, it is anticipated that the information script framed as a loss (incurred by not vaccinating the child) will increase uptake of DPT immunisation more than the information script framed as a gain (incurred by vaccinating the child).

# Study Objectives

The study will test the extent to which an intervention designed to improve household perceptions of the efficacy of preventative care increases uptake of the service in Uttar Pradesh. It will use the DPT vaccine as a case study to examine this question. The specific objectives of the study are:

1. To examine whether providing accurate information regarding the effectiveness of DPT vaccine affects demand for and uptake of the service
2. To examine whether negative framing relative to positive framing enhances the effect of the information on service uptake
3. To examine whether the effect of information is greater amongst those who initially have lower perceptions of the efficacy of DPT vaccine

The study involves a randomised field experiment to test whether information given to households on the actual effectiveness of healthcare can shift perceptions thereby increasing demand and uptake. It would only be ethical to attempt to provide households with information on health interventions for which there is rigorous evidence on efficacy. The general principle will thus be tested using an information intervention specific to the DPT vaccine for which there is strong and widely accepted evidence on efficacy.

# Methods

## Study Design

A randomised field experiment will be used to provide information on the efficacy of the DPT vaccination to mothers with young children. Families will be individually randomised into three study arms. Mothers in the first treatment group will receive the information framed in positive terms. A second treatment group will provide essentially the same information on efficacy but framed in negative terms. The third arm acts as a control group with no information given to the mother. Randomisation will done using the digital equivalent of a three-sided coin. Enumerators will be blind to the assignment of the intervention.

Information will be given to mothers in the privacy of their own home. Nevertheless, randomisation at the individual household level gives rise to concerns of potential contamination. A household given one of the two information interventions may then interact and share information with the households in the control group. The extent to which information spills over to other neighbours and peers of treated households will be estimated in the standard way, as explained in Section 4.6, by exploiting random variation in the geographical density of households.

## Participants and Inclusion Criteria

The criteria for inclusion in the study include: 1) mother with a child aged 0-36 months living in the same household; 2) child has not received three doses of DPT vaccine; 3) mother gives consent to participate in the study; and 4) mother intends to remain in the study area for at least six months. The criteria for exclusion in the study include: 1) child of mother is not aged 0-36 months; 2) child of mother is not living in the same household; 3) child has received three doses of DPT vaccine; 4) mother does not give consent to participate in the study; and 5) mother due to emigrate from the study area in the next year.

## Outcomes

The primary outcome is the proportion of children with three doses of DPT vaccine as verified by the vaccination card or reported by the mother. This outcome will be measured at baseline and nine months. Secondary outcomes include: 1) the proportion of children fully vaccinated (against tuberculosis, diphtheria, pertussis, tetanus, polio and measles); 2) an index of the mother’s knowledge of tetanus symptoms, and 3) an index of the mother’s perception of the efficacy of tetanus vaccination. Characteristics of the study population will be obtained from the household survey at baseline and used to examine the balance of the treatment and control groups.

## Sample Size Calculations

Table 3 shows the detectable difference between a single treatment group and the control group for a range of sample sizes and assumed proportions of children in the control group with three doses of DPT vaccination one year after baseline. These calculations are based on 80 percent power and 5 percent level of significance. Assuming 10 percent of children in the control group with three doses of DPT vaccination one year after baseline, the study will be able to detect a difference between a single treatment group and the control group of 8 to 9 percentage points. If the assumed proportion of children in the control group with three doses of DPT vaccination is 20 percent, then the detectable difference is slightly more than 10 percentage points. Note that the inclusion of baseline covariates should improve the precision of estimates. The second part of Table 3 shows sample size calculations when the comparison is between the two treatment groups pooled together and the control group. In this comparison, the minimum detectable difference given the sample size available to the study is approximately 7 percentage points under the assumption that 10 percent of children in the control group have three doses of DPT vaccination one year after baseline.

Table 3. Sample size calculations

|  | Effect size (percentage point difference between groups) | | | | | |
| --- | --- | --- | --- | --- | --- | --- |
|  | 5 % | 6 % | 7 % | 8 % | 9 % | 10 % |
| **1. Comparison between single intervention group and control group** | | | | | | |
| P_1_ is 10 percent | 686 | 492 | 373 | 295 | 240 | 199 |
| P_2_ is 20 percent | 1094 | 772 | 575 | 447 | 358 | 294 |
|  |  |  |  |  |  |  |
| **2. Comparison between two treatment groups pooled and control group** | | | | | | |
| P_1_ is 10 percent | 526/1052 | 379/758 | 288/576 | 228/456 | 185/370 | 155/310 |
| P_2_ is 20 percent | 829/1658 | 585/1755 | 437/874 | 340/680 | 273/546 | 224/448 |

Notes: Sample size calculations based on individual randomization, 80 percent power and 5 percent level of significance. The second part of the table examines sample sizes for a comparison of the two treatment groups and the control group showing the sample size in the control group / two treatment groups pooled. The cells highlighted indicate the approximate sample size available to the study.

## Data Collection

A number of data collection activities are envisaged as part of the overarching study evaluating the social franchise project. These data collection tools have been designed to accommodate the research objectives contained within this study protocol. In others words, this study will piggyback on the data collection activities of the overarching evaluation.

### Identification of participants

Potentially eligible participants were identified through two channels. First, a household survey conducted by the research team for a different purpose nine months prior to the study will be useful in identifying eligible women to participate. The household survey will use the sampling frame provided by the most recent census, conducted in 2011. The sampling is based on a two-stage design. The first stage involves the selection of 180 primary sampling units or clusters using systematic sampling proportional to size. The second stage involves a full household listing on entry to the cluster to create the sampling frame of eligible women (i.e. gave birth in previous two years). From this sampling frame 20 eligible women per cluster will be selected at random and invited for interview. Second, a list of mothers who have given birth in the past year provided by the community health worker (known as ASHAs) in each village will also identify eligible women to participate. Study researchers will visit the household of each potentially eligible mother, assess eligibility based on either vaccination cards or self-reports, and seek informed consent.

### Initial and final household survey

A baseline survey of eligible women will capture information on: 1) the presence of a vaccination card; 2) whether the child has been vaccinated against tuberculosis, diphtheria, pertussis, tetanus, polio and measles, respectively; 3) the mother’s knowledge of tetanus symptoms, and 4) the mother’s perceptions of the efficacy of tetanus vaccination. The latter will be measured using an established technique in which women are asked to ascertain the likelihood of various events happening by placing beans in a cup. The endline survey will use a very similar tool nine months after the baseline survey.

## Analysis

The primary analysis will compare the proportion of children with three doses of DPT in the two treatment groups pooled together against the control group. A logistic regression with a dummy variable representing the two treatment groups combined will produce risk ratios with 95% confidence intervals adjusted for the distance stratifying variable (included as two dummy variables), with the control group as the omitted reference category. Fully adjusted risk ratios will also be generated with the inclusion of baseline characteristics. Secondary outcomes will be analysed in a similar manner, but using OLS in the case of continuous outcomes.

Several additional analyses will be conducted. First, an analysis to identify the effects of the two variants of the intervention will be conducted in a treatment group analysis. Each of the three groups will be compared against each other. The comparison between the two information groups will identify whether framing matters. Second, an analysis of information spillovers will be conducted by exploiting random variation in the geographical density of households assigned the treatment groups ([Dupas, 2014](#_ENREF_5), [Kremer and Miguel, 2007](#_ENREF_12), [Miguel and Kremer, 2004](#_ENREF_19)). Information on the geographical coordinates of households will be used to generate a variable that indicates for each household the share of other households within a given radius who received the information intervention. The presence of information spillovers is examined by the inclusion of this density variable. The sensitivity of the results to different radii will be explored (ie. 250 metres, 500 metres, 750 metres). Third, differences in the primary endpoint across specified subgroups will be assessed by adding a treatment-by-subgroup interaction terms to the binary regression models. This will be done separately for the following subgroups measured at baseline: 1) index of the mother’s perception of the efficacy of tetanus vaccination; 2) gender of the child; 3) household wealth; 4) education level of the mother; and 5) distance to the nearest government facility. In the analysis of secondary outcomes, OLS will be used in the case of continuous outcomes.

# Ethics and Data Management

## Ethical and Other Approvals

Ethical clearance for this proposal has been granted by the Public Healthcare Society (PHS) Ethics Review Board in India. Ethical clearance will be sought from the London School of Hygiene and Tropical Medicine in the UK. The study protocol of the overarching study has been submitted for review by the Health Ministry Screening Committee which is given technical assistance by the Indian Council of Medical Research. Government clearance is currently being sought from the National Rural Health Mission in the State of Uttar Pradesh. Funding for the research is provided by MSD for Mothers, who have approved the study protocol.

## Ethical Considerations

The study must pose no major risk to individuals involved in the study. This is unlikely to be the case since the research requires no invasive procedures or examinations. The research activities involving data collection through the household survey are not anticipated to cause any harm. Data collection is based on standard tools that have been repeatedly implemented on different populations in numerous countries. The main cost to participants will be the time given by the interviewees. Some of the interviews with households will involve women whose baby may have recently died. The enumerators will be trained to deal with such cases sensitively. It is not anticipated that the information intervention itself will cause any harm. It will be made clear to study participants that they will be at liberty to withdraw from the study at any point without reason. Other more general ethical issues are addressed below: informed consent; processes to maintain confidentiality of the data collected (ie. secure storage of the questionnaires; anonymised data when digitised; secure storage of data including encryption), training of interviewers so that they approach the maternal health subject with sensitivity, how to handle breaches of confidentiality by members of the data collection and research teams, and appropriate dissemination of data.

## Informed Consent

Informed consent will be required from mothers before: 1) administering the household survey; and 2) delivering the information intervention. For each of three consent processes, an information form will be read and given to participants on the purpose of the study, the risks and benefits of the study, the broad content of the interview, the confidentiality of the data, the voluntary nature of the interview, the possibility of stopping the study or interview at any time without reason, and the contact details of the principal investigators. Consent from women indicated by a thumb print, with written confirmation by the enumerator of such consent being given, will be sought and must be given prior to the information intervention being delivered or the interview commencing.

## Data Management

An important ethical risk of the study pertains to breaches of confidentiality. The study will make every effort to minimise the risk of breaches of confidentiality, particularly in relation to data management and the linking of datasets. The research intends to link data from different sources. The linking of data with geographical identifiers elevates the importance of maintaining confidentiality.

A number of steps will be taken in terms of management of the data. First, data using the various data collection tools will be entered by enumerators at the time of interview and will be linked only by the PIs for the purposes of random assignment and analysis. In this way, misuse or breach of confidentiality at the data entry level will be reduced by a great extent. Second, data will be stored electronically (no paper questionnaires are to be used) in London (at LSHTM) and Delhi (at Sambodhi) and will only be accessed by the PIs and other members of the research team. No other persons will have access to the securely kept data.

Third, the datasets will contain no information that would allow identification of any individual woman or household. They will contain no information on names or GPS information. The village cluster will be coded such that it is not possible to know the name of the village from the datasets. GPS information on households will be collected using a separate data collection form, entered into digital format separately and the digital format of the data will be stored in its own data file. Datasets will not be linked to the GPS information dataset, except by the PIs at the time of analysis. The variables containing the GPS information will otherwise be scrambled such there is an uncertainty in the location of observation of at least 10 kilometres.

Fourth, as is common practice, data will be reported in aggregate format only (eg. means, subgroup means, percentages, regression outputs) such that no individual is identified. Finally, at the end of the project the data will be made available to researchers under conditions of restricted access. The data can only be used for research or teaching purposes. Access the data will be granted by the PIs on an individual basis in response to requests made using a form. The data will not contain any identifiers or names that would allow identification of an individual. The platform to make the data available to researchers will be the LSHTM data repository.

# References

ASHRAF, N., JACK, B. K. & KAMENICA, E. 2013. Information and subsidies: Complements or substitutes? *Journal of Economic Behavior & Organization,* 88**,** 133-139.

BERTRAND, M., KARLAN, D., MULLAINATHAN, S., SHAFIR, E. & ZINMAN, J. 2010. What's Advertising Content Worth? Evidence from a Consumer Credit Marketing Field Experiment. *Quarterly Journal of Economics,* 125**,** 263-306.

BHUTTA, Z. A., CHOPRA, M., AXELSON, H., BERMAN, P., BOERMA, T., BRYCE, J., BUSTREO, F., CAVAGNERO, E., COMETTO, G., DAELMANS, B., DE FRANCISCO, A., FOGSTAD, H., GUPTA, N., LASKI, L., LAWN, J., MALIQI, B., MASON, E., PITT, C., REQUEJO, J., STARRS, A., VICTORA, C. G. & WARDLAW, T. 2010. Countdown to 2015 decade report (2000-10): taking stock of maternal, newborn, and child survival. *Lancet,* 375**,** 2032-44.

CAIRNCROSS, S., SHORDT, K., ZACHARIA, S. & GOVINDAN, B. K. 2005. What causes sustainable changes in hygiene behaviour? A cross-sectional study from Kerala, India. *Social Science & Medicine,* 61**,** 2212-2220.

DUPAS, P. 2014. Short-Run Subsidies and Long-Run Adoption of New Health Products: Evidence from a Field Experiment. *Econometrica,* 82**,** 197-228.

GIVORD, P. & ROMANELLO, L. 2011. Evaluation of a Community-based Information Campaign on Health Demand in Mali: Results from a Natural Experiment.

GODLONTON, S., MUNTHALI, A. & THORNTON, R. 2011. Behavioral response to information? Circumcision, information, and HIV prevention. University of Michigan Working Paper.

JALAN, J. & SOMANATHAN, E. 2008. The importance of being informed: Experimental evidence on demand for environmental quality. *Journal of Development Economics,* 87**,** 14-28.

JAMISON, D. T. 2006. Investing in Health. *In:* JAMISON, D. T., BREMAN, J. G., MEASHAM, A. R., ALLEYNE, G., CLAESON, M., EVANS, D. B., JHA, P., MILLS, A. & MUSGROVE, P. (eds.) *Disease Control Priorities in Developing Countries.* 2nd ed. Washington (DC).

JONES, G., STEKETEE, R. W., BLACK, R. E., BHUTTA, Z. A., MORRIS, S. S. & BELLAGIO CHILD SURVIVAL STUDY, G. 2003. How many child deaths can we prevent this year? *Lancet,* 362**,** 65-71.

KAHNEMAN, D. & TVERSKY, A. 1979. Prospect Theory: An Analysis of Decision under Risk. *Econometrica,* 47**,** 263-91.

KREMER, M. & MIGUEL, E. 2007. The Illusion of Sustainability. *Quarterly Journal of Economics,* 122**,** 1007-65.

LIU, L., OZA, S., HOGAN, D., PERIN, J., RUDAN, I., LAWN, J. E., COUSENS, S., MATHERS, C. & BLACK, R. E. 2014. Global, regional, and national causes of child mortality in 2000-13, with projections to inform post-2015 priorities: an updated systematic analysis. *Lancet*.

LUCAS, P. J., CABRAL, C. & COLFORD JR, J. M. 2011. Dissemination of drinking water contamination data to consumers: A systematic review of impact on consumer behaviors. *PloS one,* 6**,** e21098.

LUO, R., SHI, Y., ZHANG, L., ZHANG, H., MILLER, G., MEDINA, A. & ROZELLE, S. 2012. The limits of health and nutrition education: Evidence from three randomized-controlled trials in rural China. *CESifo Economic Studies,* 58**,** 385-404.

LUOTO, J., LEVINE, D. & ALBERT, J. 2011. Information and Persuasion Achieving Safe Water Behaviors in Kenya *In:* RAND (ed.) *RAND Labor and Population working paper series.*

MADAJEWICZ, M., PFAFF, A., VAN GEEN, A., GRAZIANO, J., HUSSEIN, I., MOMOTAJ, H., SYLVI, R. & AHSAN, H. 2007. Can information alone change behavior? Response to arsenic contamination of groundwater in Bangladesh. *Journal of Development Economics,* 84**,** 731-754.

MEREDITH, J., ROBINSON, J., WALKER, S. & WYDICK, B. 2013. Keeping the doctor away: Experimental evidence on investment in preventative health products. *Journal of Development Economics,* 105**,** 196-210.

MIGUEL, E. & KREMER, M. 2004. Worms: Identifying Impacts on Education and Health in the Presence of Treatment Externalities. *Econometrica,* 72**,** 159-217.

MILLION DEATH STUDY, C., BASSANI, D. G., KUMAR, R., AWASTHI, S., MORRIS, S. K., PAUL, V. K., SHET, A., RAM, U., GAFFEY, M. F., BLACK, R. E. & JHA, P. 2010. Causes of neonatal and child mortality in India: a nationally representative mortality survey. *Lancet,* 376**,** 1853-60.

MONTAGU, D. 2002. Franchising of health services in low-income countries. *Health policy and planning,* 17**,** 121-30.

RHEE, M., SISSOKO, M., PERRY, S., MCFARLAND, W., PARSONNET, J. & DOUMBO, O. 2005. Use of insecticide-treated nets (ITNs) following a malaria education intervention in Piron, Mali: a control trial with systematic allocation of households. *Malaria Journal,* 4**,** 35.

ROTHMAN, A. J. & KIVINIEMI, M. T. 1999. Treating people with information: an analysis and review of approaches to communicating health risk information. *JNCI Monographs,* 1999**,** 44-51.

SANDERS, D. & HAINES, A. 2006. Implementation research is needed to achieve international health goals. *PLoS Med,* 3**,** e186.

SINGH, P. 2011. Performance Pay and Information: Reducing Child Malnutrition in Urban Slums.

TAKASAKI, Y. & SATO, R. 2013. Why don’t the poor receive antenatal care?: Evidence from incentive and information experiments in rural Nigeria.

TAROZZI, A., BENNEAR, L., BALASUBRAMANYA, S., PFAFF, A., MATIN, A. & VAN GEEN, L. 2010. Subjective Risk Assessment and Reactions to Health-related Information: Evidence from Bangladesh.

TRAVIS, P., BENNETT, S., HAINES, A., PANG, T., BHUTTA, Z., HYDER, A. A., PIELEMEIER, N. R., MILLS, A. & EVANS, T. 2004. Overcoming health-systems constraints to achieve the Millennium Development Goals. *Lancet,* 364**,** 900-6.

TVERSKY, A. & KAHNEMAN, D. 1991. Loss Aversion in Riskless Choice: A Reference-Dependent Model. *Quarterly Journal of Economics,* 106**,** 1039-61.

VICTORA, C. G., BARROS, A. J., AXELSON, H., BHUTTA, Z. A., CHOPRA, M., FRANCA, G. V., KERBER, K., KIRKWOOD, B. R., NEWBY, H., RONSMANS, C. & BOERMA, J. T. 2012. How changes in coverage affect equity in maternal and child health interventions in 35 Countdown to 2015 countries: an analysis of national surveys. *Lancet,* 380**,** 1149-56.

# Appendix

## Updates to the original study protocol

A number of changes were made to the original study protocol (dated 3rd December 2014) before the baseline household survey and the delivery of the information intervention. These changes are reflected in both this version of the study protocol and the online trial registration for the study. The changes and the justification for the changes were as follows:

- Eligible mothers were randomly assigned in a ratio of 1:1:1 to one of three study arms using simple randomisation. In the original study protocol it stated that randomisation would be stratified according to distance of the residence to the nearest public health facility. We did not collect data on distance to the nearest public health facility because we felt such information would be unreliable.
- Potentially eligible participants were identified through two channels: a household survey conducted by the research team nine months prior to the study and a list of mothers who had given birth in the past year provided by the community health worker (known as ASHAs) in each village. In the original study protocol it stated that all participants would be identified through the first channel only. After collecting data from the household survey conducted nine months prior to the start of the study, it became clear the sample sizes obtainable from this method would be insufficient. Hence, the second channel of identifying eligible participants was also used.
- One criteria for inclusion in the study was that the child should be aged 0-36 months. In the original study protocol the age range was 0-11 months. Revisions to the study protocol recognised that older children should be eligible because they can receive catch-up vaccinations for DPT.
- In the original study protocol we envisaged collecting data on general health care seeking behaviour of members in the participating households using a family health pictorial diary. This data collection method was dropped altogether.

## Deviations from the study protocol

There were deviations from the study protocol as follows:

- Before the baseline household survey and the delivery of the information intervention, the secondary outcome measuring the proportion of children fully vaccinated was re-defined to no longer include polio, as originally stated in the study protocol. We did not collect any information on polio vaccinations in the household surveys.
- Prior to the data analysis, we planned to analyse the following additional secondary outcomes: the proportion of children with measles vaccination, the proportion of children with BCG, the mother’s knowledge of any cause of tetanus and her knowledge of any prevention method.
- In the study protocol, it stated that we would conduct subgroup analyses with respect to: 1) index of the mother’s perception of the efficacy of tetanus vaccination; 2) gender of the child; 3) household wealth; 4) education level of the mother; and 5) distance to the nearest government facility. The last three subgroup analyses were not conducted because we did not collect data on household wealth, education or distance to the nearest government health facility.
- The original protocol stated that a logistic regression would be used to produce odd ratios of the effect of the information intervention. Since simple randomisation was used that involved no stratification, we resorted to basic statistics to show the absolute difference and the relative risk between groups, with their corresponding 95% confidence intervals.
- We conducted several analyses that were unplanned. We decided to do these analyses after we had completed the analysis of the pre-specified outcomes, hence they are exploratory. First, we compared the mean in our measure of perception of DPT vaccine efficacy between treatment and control for two subgroups of women, with baseline perception of efficacy above and below the 50% threshold, respectively. Second, we carried out a cost-effectiveness analysis.
