# Supplementary material for: Effect and cost-effectiveness of educating mothers about childhood DPT vaccination on immunisation uptake, knowledge, and perceptions in Uttar Pradesh, India: A randomised controlled trial
Source: PLoS Med. 2018 Mar 6;15(3):e1002519. doi: 10.1371/journal.pmed.1002519 (PMC5839535; doi:10.1371/journal.pmed.1002519)
Supplement: S1 Table — Values are n/N (%) for binary outcomes and mean (standard deviation) for continuous outcomes. None of the differences between groups are significant at the 5% level. Scheduled caste and scheduled tribe are among the most disadvantaged socioeconomic groups in India. Other backward caste is an additional category of socially and economically disadvantaged people. All 3 are official terms used by the government of India and given explicit recognition in India’s constitution. (DOCX) [file pmed.1002519.s007.docx]

|  | Control | |  | Positive | |  | Negative | |
| --- | --- | --- | --- | --- | --- | --- | --- | --- |
| Outcomes | Freq (%), Mean (sd) | |  | Freq (%), Mean (sd) | |  | Freq (%), Mean (sd) | |
| Scheduled caste | 54/153 (35%) | |  | 56/161 (35%) | |  | 50/145 (34%) | |
| Scheduled tribe | 7/153 (5%) | |  | 7/161 (4%) | |  | 2/145 (1%) | |
| Other backward caste | 68/153 (44%) | |  | 80/161 (50%) | |  | 73/145 (50%) | |
| General caste | 23/153 (15%) | |  | 18/161 (11%) | |  | 17/145 (12%) | |
| Hindu | 135/153 (88%) | |  | 130/161 (81%) | |  | 122/145 (84%) | |
| Muslim | 18/153 (12%) | |  | 31/161 (19%) | |  | 23/145 (16%) | |
| Urban | 33/153 (22%) | |  | 24/161 (15%) | |  | 30/145 (21%) | |
| Household size | 5.64 (0.16) | |  | 5.44 (0.15) | |  | 5.78 (0.20) | |
| Number of rooms | 1.67 (0.06) | |  | 1.56 (0.05) | |  | 1.8 (0.09) | |
| Hh wealth quintile | 2.88 (0.12) | |  | 2.73 (0.11) | |  | 2.70 (0.12) | |
| Hh has health insurance | 9/151 (6%) | |  | 14/160 (9%) | |  | 18/143 (13%) | |
| Age of mother | 26.54 (0.39) | |  | 26.22 (0.34) | |  | 26.25 (0.38) | |
| Household's education | 6.07 (0.39) | |  | 6.59 (0.37) | |  | 5.80 (0.39) | |
| Mother's education | 5.70 (0.42) | |  | 4.98 (0.39) | |  | 5.41 (0.45) | |
|  |  |  |  |  |  |  |  |  |
| N | 153 | |  | 161 | |  | 145 | |
