# Supplementary material for: Effect and cost-effectiveness of educating mothers about childhood DPT vaccination on immunisation uptake, knowledge, and perceptions in Uttar Pradesh, India: A randomised controlled trial
Source: PLoS Med. 2018 Mar 6;15(3):e1002519. doi: 10.1371/journal.pmed.1002519 (PMC5839535; doi:10.1371/journal.pmed.1002519)
Supplement: S2 Table — All regressions are ordinary least squares, adjusted for child age and baseline measure of the outcome. Treatment effects are in terms of absolute differences. Results from the 2 analyses are presented: pooled analysis and treatment group analysis. (DOCX) [file pmed.1002519.s008.docx]

| Outcomes | Pooled analysis  Difference (95% CI) | | |  | | Treatment group analysis  Difference (95% CI) | | | | | | | | | | | |
| --- | --- | --- | --- | --- | --- | --- | --- | --- | --- | --- | --- | --- | --- | --- | --- | --- | --- |
|  | T vs C | | |  | | Pos vs C | | | | Neg vs C | | | | Neg vs Pos | | | |
| Primary outcome |  |  | |  | |  | |  | |  | |  | |  | |  | |
| DPT3 vaccine | 0.148 (0.08-0.22) | | |  | | 0.130 (0.05 to 0.21) | | | | 0.165 (0.08 to 0.25) | | | | 0.036 (-0.05 to 0.12) | | | |
| DPT3 vaccination card | 0.127 (0.02-0.23) | | |  | | 0.124 (-0.00 to 0.25) | | | | 0.130 (0.01 to 0.25) | | | | 0.009 (-0.11 to 0.13) | | | |
| DPT3 self-reported | 0.134 (0.05-0.22) | | |  | | 0.119 (0.03 to 0.21) | | | | 0.153 (0.06 to 0.25) | | | | 0.030 (-0.08 to 0.14) | | | |
| Secondary outcomes |  |  |  | |  | |  | |  | |  | |  | |  | |  |
| Fully vaccinated | 0.145 (0.08-0.22) | | |  | | 0.127 (0.05 to 0.21) | | | | 0.163 (0.09 to 0.24) | | | | 0.038 (-0.05 to 0.12) | | | |
| BCG vaccine | 0.007 (-0.03-0.04) | | |  | | 0.007 (-0.03 to 0.04) | | | | 0.010 (-0.03 to 0.05) | | | | 0.006 (-0.03 to 0.05) | | | |
| Measles vaccine | 0.226 (0.16-0.30) | | |  | | 0.222 (0.14 to 0.30) | | | | 0.231 (0.15 to 0.31) | | | | 0.009 (-0.08 to 0.01) | | | |
| Knowledge of causes | 0.381 (0.32-0.50) | | |  | | 0.369 (0.30 to 0.44) | | | | 0.399 (0.33 to 0.47) | | | | 0.035 (-0.03 to 0.01) | | | |
| Knowledge of symptoms | 0.387 (0.32-0.46) | | |  | | 0.384 (0.31 to 0.46) | | | | 0.391 (0.32 to 0.47) | | | | 0.010 (-0.08 to 0.01) | | | |
| Knowledge of prevention | 0.257 (0.19-0.32) | | |  | | 0.208 (0.13 to 0.29) | | | | 0.307 (0.23 to 0.38) | | | | 0.100 (0.03 to 0.18) | | | |
| Perception of efficacy | 0.188 (-0.14-0.52) | | |  | | 0.072 (-0.31 to 0.45) | | | | 0.318 (-0.06 to 0.70) | | | | 0.260 (-0.13 to 0.65) | | | |
