# Supplementary material for: Effect and cost-effectiveness of educating mothers about childhood DPT vaccination on immunisation uptake, knowledge, and perceptions in Uttar Pradesh, India: A randomised controlled trial
Source: PLoS Med. 2018 Mar 6;15(3):e1002519. doi: 10.1371/journal.pmed.1002519 (PMC5839535; doi:10.1371/journal.pmed.1002519)
Supplement: S3 Table — (DOCX) [file pmed.1002519.s009.docx]

| Outcomes | Difference (unadjusted p value) | | |  | Difference (Bonferroni adjusted p value) | | |
| --- | --- | --- | --- | --- | --- | --- | --- |
|  | Pos vs C | Neg vs C | Neg vs Pos |  | Pos vs C | Neg vs C | Neg vs Pos |
| Primary Outcome |  |  |  |  |  |  |  |
| DPT3 combined | 0.124 | 0.167 | 0.043 |  | 0.124 | 0.167 | 0.043 |
|  | (0.005) | (<0.001) | (0.352) |  | (0.014) | (<0.001) | (1.000) |
| DPT3 vaccination card | 0.148 | 0.193 | 0.044 |  | 0.148 | 0.193 | 0.044 |
|  | (0.035) | (0.005) | (0.513) |  | (0.106) | (0.015) | (1.000) |
| DPT3 self-reported | 0.117 | 0.146 | 0.030 |  | 0.117 | 0.146 | 0.030 |
|  | (0.014) | (0.003) | (0.580) |  | (0.043) | (0.008) | (1.000) |
