# Supplementary material for: Effect and cost-effectiveness of educating mothers about childhood DPT vaccination on immunisation uptake, knowledge, and perceptions in Uttar Pradesh, India: A randomised controlled trial
Source: PLoS Med. 2018 Mar 6;15(3):e1002519. doi: 10.1371/journal.pmed.1002519 (PMC5839535; doi:10.1371/journal.pmed.1002519)
Supplement: S4 Table — All regressions are ordinary least squares. The 2 information groups are pooled together as a single treatment group. Standard errors are presented in parentheses. The share of treated neighbours is the number of other study participants who were assigned to receive the information intervention divided by total number of study participants within a specified radius. * p < 0.10, ** p < 0.05, *** p < 0.01. (DOCX) [file pmed.1002519.s010.docx]

|  | (1) | (2) | (3) | (4) | (5) | (6) |
| --- | --- | --- | --- | --- | --- | --- |
|  |  | DPT3 |  |  |  |  |
| Variables | DPT3 | cluster fixed effects | DPT3 | DPT3 | DPT3 | DPT3 |
|  |  |  |  |  |  |  |
| Information | 0.149*** | 0.169*** | 0.149*** | 0.115 | 0.149*** | 0.147*** |
|  | (0.0384) | (0.0457) | (0.0385) | (0.0827) | (0.0385) | (0.0384) |
| Share of treated neighbours within 250m | -0.0796 | -0.0806 | -0.0869 | -0.118 |  |  |
|  | (0.0558) | (0.0944) | (0.0574) | (0.0994) |  |  |
| Share of treated neighbours within 500m |  |  |  |  | -0.0695 |  |
|  |  |  |  |  | (0.0601) |  |
| Share of treated neighbours within 1km |  |  |  |  |  | -0.0817 |
|  |  |  |  |  |  | (0.0660) |
| Total number of neighbours in 250m |  |  | 0.00397 |  |  |  |
|  |  |  | (0.00701) |  |  |  |
| Share of treated neighbours in 250m * information |  |  |  | 0.0563 |  |  |
|  |  |  |  | (0.120) |  |  |
| Constant | 0.328*** | 0.316*** | 0.316*** | 0.351*** | 0.323*** | 0.333*** |
|  | (0.0457) | (0.0797) | (0.0507) | (0.0670) | (0.0478) | (0.0523) |
| Observations | 706 | 706 | 706 | 706 | 706 | 706 |
| R-squared | 0.023 | 0.039 | 0.023 | 0.023 | 0.022 | 0.022 |
