# Supplementary material for: Effect and cost-effectiveness of educating mothers about childhood DPT vaccination on immunisation uptake, knowledge, and perceptions in Uttar Pradesh, India: A randomised controlled trial
Source: PLoS Med. 2018 Mar 6;15(3):e1002519. doi: 10.1371/journal.pmed.1002519 (PMC5839535; doi:10.1371/journal.pmed.1002519)
Supplement: S5 Table — Sources are the Indian Census 2011 and the Annual Health Survey 2012–13. The maternal mortality ratio estimates apply to groups of districts within the state due to sample size limitations. (DOCX) [file pmed.1002519.s011.docx]

| Indicator | Uttar Pradesh | Kannauj | Kanpur Nagar | Kanpur Dehat | Auraiya | Etawah | Fatehpur |
| --- | --- | --- | --- | --- | --- | --- | --- |
| Population (in millions | 199.8 | 1.7 | 4.6 | 1.8 | 1.4 | 1.6 | 2.6 |
| Rural population (%) | 78 | 83 | 34 | 90 | 83 | 77 | 88 |
| Literacy (%) | 57 | 61 | 71 | 65 | 67 | 67 | 57 |
| Scheduled caste (%) | 21 | 19 | 18 | 26 | 28 | 25 | 25 |
| Scheduled tribe (%) | 0.57 | < 0.1 | < 0.1 | < 0.1 | < 0.1 | < 0.1 | < 0.1 |
| Fertility (lifetime) | 3.3 | 3.3 | 2.1 | 2.8 | 3.5 | 3.1 | 3.5 |
| Maternal mortality ratio (per 100,000) | 258 | 240 | 240 | 240 | 240 | 240 | 283 |
| Under five mortality (per 1,000) | 90 | 102 | 50 | 94 | 84 | 85 | 81 |
| Infant mortality (per 1,000) | 68 | 79 | 37 | 65 | 58 | 56 | 55 |
| Neonatal mortality (per 1,000) | 49 | 55 | 24 | 41 | 41 | 41 | 39 |
|  | | | | | | | |
