# Supplementary material for: Effect and cost-effectiveness of educating mothers about childhood DPT vaccination on immunisation uptake, knowledge, and perceptions in Uttar Pradesh, India: A randomised controlled trial
Source: PLoS Med. 2018 Mar 6;15(3):e1002519. doi: 10.1371/journal.pmed.1002519 (PMC5839535; doi:10.1371/journal.pmed.1002519)
Supplement: S1 Text — (DOCX) [file pmed.1002519.s012.docx]

# S1 Text. Study setting

Uttar Pradesh is India’s most populous state with 199.8 million people living in 18 divisions and 75 districts in 2011. The proportion of the population living in rural areas is 77%. Over half of the population is literate and 31% live below the poverty line. In 2010-2011, Uttar Pradesh’s total fertility rate was an estimated 3.6, and the median age at first live birth for women between 15-49 years is 22 years. Maternal and infant mortality remain high, with most recent estimates of the MMR at 258 deaths per 100,000 live births and infant mortality at 68 deaths per 1000 live births (S5 Table) [[1](#_ENREF_1),[2](#_ENREF_2),[3](#_ENREF_3)].

The study was located in six districts of Uttar Pradesh: Kannuaj, Kanpur Nagar, Kanpur Dehat, Auraiya, Etawah, and Fatehpur. These districts vary only modestly in terms of demographic and health indicators, except for one clear outlier (S5 Table). Kanpur Nagar is predominantly urban, with higher literacy and lower mortality than the state average. By contrast, the other districts are more typical of the state as a whole. Largely rural, they have poor literacy and high rates of maternal and child mortality that are comparable with the less developed countries in the world.

# References

1. Office of the Registrar General (2013) Special Bulletin on Maternal Mortality in India 2010-12. New Delhi, India: Government of India.

2. Office of the Registrar General & Census Commissioner (2012-2013) Annual Health Survey 2012-2013 Fact Sheet, Uttar Pradesh. New Delhi, India: Government of India.

3. Office of the Registrar General & Census Commissioner (2013) Census of India 2011. New Delhi, India: Government of India.
