# Supplementary material for: Effect and cost-effectiveness of educating mothers about childhood DPT vaccination on immunisation uptake, knowledge, and perceptions in Uttar Pradesh, India: A randomised controlled trial
Source: PLoS Med. 2018 Mar 6;15(3):e1002519. doi: 10.1371/journal.pmed.1002519 (PMC5839535; doi:10.1371/journal.pmed.1002519)
Supplement: S2 Text — (DOCX) [file pmed.1002519.s013.docx]

# S2 Text. Sampling strategy

The information study was embedded within a broader research project that involved data collection in 180 clusters across the six study districts [1]. The broader research project involved the evaluation of social franchising model in three districts in Uttar Pradesh. The three districts in which the social franchise was rolled out were selected by the programme implementers. We additionally selected three neighbouring districts out of a possible nine neighbouring districts. The three neighbouring districts were selected to be as similar as possible to the intervention districts based on the following district level indicators obtained from the Annual Health Survey: % urban population; % literate; total fertility rate; median age; % modern contraception; % ANC four visits; % deliver in government facility; % deliver in private facility; % fully immunised.

The social franchise study involved the selection of three types of clusters: (1) intervention clusters with a Sky provider; (2) internal comparison clusters with no social franchisee in the three intervention districts; and (3) external comparison clusters in three neighbouring districts where the social franchise model was not operating [2]. We selected study clusters 1 year after the first social franchisee was contracted with the following procedures. First, every Sky health provider was linked to its census area. 393 private providers were in the network at the time of selection, with membership reflecting decisions by the providers on whether to join and by the franchiser on which providers to target. The process of linking Sky providers to census areas led to the identification of 216 possible intervention clusters from which we selected 60 clusters at random. Second, we selected internal comparison clusters by matching without replacement the intervention clusters to 60 comparison areas within the same three districts. We used census data on village characteristics for the matching. To limit problems of contamination, we did not select comparison clusters adjacent to intervention areas. Finally, we did the same matching procedure to select 60 external comparison clusters from neighbouring districts. While the study clusters were not selected to be representative of the six study districts, census data suggest that they were demographically similar to the study districts and the state (Fig S1).

# References

1. S K. Pereira, P Kumar, V Dutt, et al. (2015) Protocol for the evaluation of a social franchising model to improve maternal health in Uttar Pradesh, India. Implement Science 10: 77.

2. Tougher S, Dutt V, Pereira S, et al. (2017) Effect of a multifaceted social franchising model on quality and coverage of maternal, newborn, and reproductive health-care services in Uttar Pradesh, India: a quasi-experimental study. Lancet Glob Health.
