# Supplementary material for: Effect and cost-effectiveness of educating mothers about childhood DPT vaccination on immunisation uptake, knowledge, and perceptions in Uttar Pradesh, India: A randomised controlled trial
Source: PLoS Med. 2018 Mar 6;15(3):e1002519. doi: 10.1371/journal.pmed.1002519 (PMC5839535; doi:10.1371/journal.pmed.1002519)
Supplement: S3 Text — (DOCX) [file pmed.1002519.s014.docx]

# S3 Text. Method to measure perceived efficacy of DPT vaccine

We used games with chickpeas and kidney peas to elicit responses on mothers’ perceived efficacy of the DPT vaccine. Mothers were first given the following introductory information: “Now I am going to ask you a few questions using Kidney Beans (Rajma) and Chickpeas (Chana). There are no right or wrong answers. I just want to know your opinion. Imagine there are two villages close to each other. Each village has 10 children. The kidney beans represent the children in village A and the chickpeas are the children in village B. In village A no child has received the tetanus (DPT) vaccine while all 10 children in village B have received the recommended three doses of the vaccine.”

They were then asked a series of questions. Mothers were first asked to answer a question about the probability of infection under a full coverage immunisation scenario (question 301) and under a zero-coverage immunisation scenario (question 302):

- Q301: “Using the kidney beans, can you tell me how many children in Village A are likely to fall sick with tetanus disease within one year?”
- Q302: “Now using the chickpeas, can you tell me how many children in Village B are likely to fall sick with tetanus disease within one year?” PROMPT: Remember no child in Village A has received DPT vaccine, while all ten children in village B have received the vaccine

The purpose of these questions was to introduce mothers to the general idea behind the questions and help them understand the game. We expected mothers who understood the game to give a higher answer to question 301 than to 302. In other words, this provided us with a check on whether the mother understood the logic of the game.

Mothers were then asked to imagine a hypothetical situation in which all 10 children in the village with zero coverage of DPT vaccine fell sick with tetanus, as follows:

- Q303: “Suppose in this year, we find out that in fact all 10 children in village A fell sick with tetanus. Using the chickpeas can you tell me how many children in village B are likely to have fallen sick from tetanus during the same year? PROMPT: Remember no child in Village A has received DPT vaccine, while all ten children in village B have received the vaccine

The number of chickpeas laid down in response to question 303 provided the basis to generate our measure of perceived efficacy. Responses were reverse scored – for example, an answer of 2 chickpeas (out of 10) implied a perceived efficacy of 80%, an answer of 0 chickpeas implied a perceived efficacy of 100% and so on.
