# Supplementary material for: Effect and cost-effectiveness of educating mothers about childhood DPT vaccination on immunisation uptake, knowledge, and perceptions in Uttar Pradesh, India: A randomised controlled trial
Source: PLoS Med. 2018 Mar 6;15(3):e1002519. doi: 10.1371/journal.pmed.1002519 (PMC5839535; doi:10.1371/journal.pmed.1002519)
Supplement: S4 Text — (DOCX) [file pmed.1002519.s015.docx]

# S4 Text. Cost-effectiveness calculations

We conducted cost-effectiveness analyses under two scenarios: 1) actual project implementation and 2) a scale-up of the information intervention to the entire target population in the six study districts. The scale-up scenario made different assumptions about the costs but did not change any assumptions regarding the health impacts. Health benefits under both scenarios were calculated in terms of disability-adjusted life years (DALY) using using a period life expectancy of 67.8 years at two years of age from the Indian Census 2011 [1] and applying a discount rate of 3% to future years of life, such that each child death averted corresponds to 28.87 DALYs.

Child deaths averted were calculated by multiplying the number of additional children who were vaccinated due to the intervention by the reduction in disease-specific under 5 mortality due to the vaccines. The number of additional children who were vaccinated was obtained by applying the impact estimates of DPT3 (14.6 percentage points) and measles (22 percentage points) to the total treated population. The intervention resulted in 69 additional children with three doses of DPT vaccine and 104 additional children immunised against measles.

Calculations of the reduction in under mortality required estimates of the baseline mortality rates among unvaccinated children. These estimates were based on the rates of disease-specific under 5 mortality reported by Morris S.K. et al [2] for measles (6.1 deaths per 1,000 live births due to measles) and by WHO [3] for pertussis and tetanus (0.7 deaths per 1,000 live births due to pertussis; 0 deaths per 1,000 live births due to tetanus). We adjusted these population level under 5 mortality rates to reflect the fact that the information intervention targeted unvaccinated children. The logic was that mortality rates for vaccine preventable diseases are higher amongst unvaccinated children than the general population. We inflated baseline disease-specific mortality rates for unvaccinated children using information on the efficacy of the corresponding vaccines (95% for measles and 84% for pertussis) [4,5] and the share of unvaccinated children in the population. Reductions in disease-specific U5 mortality for children vaccinated as a result of the information intervention were calculated as the baseline mortality rate multiplied by the efficacy of the vaccine (95% for measles and 84% for pertussis).

In the scale-up scenario we considered the expansion of the intervention to all unvaccinated children aged 12-23 months in the six study districts. To estimate the total number of unvaccinated children aged 12-23 months in the study areas we exploited the demographic projection package DemProj of the Lives Saved Tool (LiST) [6] using the 2011 India Census data. The projection resulted in a total of 358,150 children aged 12-23 months in the six districts in 2016, assuming no internal migration and a total fertility rate declining by 0.05 each year from 3.05 in 2016. Using initial coverage estimates obtained from the pre-baseline household survey we calculated that in 2016 the total number of children who would have not received three doses of DPT vaccine corresponds to 204,145. This is the target population we used to cost the scale-up scenario. With 63 field staff per district, each working 180 days, we calculated that the overall scale-up cost would be $2,507,616.63. This scenario assumed that each staff member would be able to reach on average 6 households per day in a village, which is about double the number of households that were reached during actual implementation. We believe this is a realistic assumption, since during the actual implementation the number of households reached was dictated by sample sizes considerations. This meant field staff were in fact under-utilised in the sense that they could have given information to more households in a given village had they been instructed to do so. All other unit costs were the same as in the actual project implementation scenario. The mean cost of the intervention would be $12.28 per beneficiary, $84.13 per additional child with DPT3, $55.83 per additional child with measles vaccine, $94.56 per DALY averted, and $2,840 per under-five death averted under this scale-up scenario.

# References

1. Office of the Registrar General & Census Commissioner I-CoI SRS Based Life Table 2011-2015.

2. Morris S, Awasthi S, Kumar R, et al. (2013) Measles mortality in high and low burden districts of India: Estimates from a nationally representative study of over 12,000 child deaths. Vaccine (2013).

3. WHO (2016) Global Health Observatory data repository: distribution of causes of death among children aged <5 years. Geneva: World Health Organization.

4. Sudfeld CR, Navar AM, Halsey NA (2010) Effectiveness of measles vaccination and vitamin A treatment. International Journal of Epidemiology.

5. Fulton T, Phadke V, Orenstein W, Hinman A, Johnson W, Omer S (2016) Protective Effect of Contemporary Pertussis Vaccines: A Systematic Review and Meta-analysis. Clinical Infectious Diseases Vol 62, Issue 9: 1100-1110.

6. Walker N TY, Friberg IK (2013) Overview of the Lives Saved Tool (LiST). BMC Public Health 13 Suppl 3:S1.
